# Extracellular matrix stiffness mediates uterine repair via the Rap1a/ARHGAP35/RhoA/F-actin/YAP axis

**DOI:** 10.1186/s12964-022-01018-8

**Published:** 2023-01-23

**Authors:** Tao Zhang, Ruiting Hu, Yan Wang, Shuai Guo, Zhimin Wu, Junfeng Liu, Chunyang Han, Changwei Qiu, Ganzhen Deng

**Affiliations:** 1 College of Animal Science and Technology, Anhui Agricultural University, Hefei, 230031 People’s Republic of China; 2 Department of Clinical Veterinary Medicine, College of Veterinary Medicine, Huazhong Agricultural University, Wuhan, 430070 People’s Republic of China; 3 College of Animal Science and Technology, Tarim University, Alar, 843300 Xinjiang People’s Republic of China

**Keywords:** Mechanotransduction, Hippo-YAP, Uterine repair, ECM stiffness, Rap1a

## Abstract

The integrity of the structure and function of the endometrium is essential for the maintenance of fertility. However, the repair mechanisms of uterine injury remain largely unknown. Here, we showed that the disturbance of mechanical cue homeostasis occurs after uterine injury. Applying a multimodal approach, we identified YAP as a sensor of biophysical forces that drives endometrial regeneration. Through protein activation level analysis of the combinatorial space of mechanical force strength and of the presence of particular kinase inhibitors and gene silencing reagents, we demonstrated that mechanical cues related to extracellular matrix rigidity can turn off the Rap1a switch, leading to the inactivation of ARHGAP35and then induced activation of RhoA, which in turn depends on the polymerization of the agonist protein F-actin to activate YAP. Further study confirmed that mechanotransduction significantly accelerates remodeling of the uterus by promoting the proliferation of endometrial stromal cells in vitro* and *in vivo. These studies provide new insights into the dynamic regulatory mechanisms behind uterine remodeling and the function of mechanotransduction.

**Video Abstract**

**Supplementary Information:**

The online version contains supplementary material available at 10.1186/s12964-022-01018-8.

## Introduction

The mammalian endometrium is a tissue that undergoes dynamic remodeling; that is, its luminal epithelial cells (LE) and glandular epithelium (GE) undergo cyclical proliferation and are then physically shed under the regulation of physiological factors such as fluctuating levels of sex steroid hormones. For women, the endometrium thickens by 4–10 mm under the influence of circulating ovarian hormones within ~ 1 week during each menstrual cycle (~ 28 days) during the woman’s reproductive lifespan (~ 400–500 uterine menstrual cycles) [1, 2]. In nonmenstruating species (e.g., mice), endometrial epithelial cells undergo rounds of proliferation (approximately ninefold) and apoptosis during the estrous cycle [3]. This tight regulation of the periodic uterine regeneration mechanism is absolutely essential for female reproductive function and general health. Aberrant regeneration of the endometrium leads to a range of uterine diseases, including endometriosis, endometrial hyperplasia, infertility, and endometrial cancer [4, 5].

Tissues and cells within organisms are continuously exposed to complex mechanical cues from the environment. Mounting evidence over the past two decades has established that mammalian cells are surrounded by extracellular matrix (ECM) and neighboring cells that provide cells with structural support and mechanical cues that affect fundamental cellular processes, including spreading, growth, proliferation, migration, differentiation and organoid formation [6–9]. ECM elasticity, or stiffness, which is central to tissue shaping, 3-D tissue architecture and organoid formation, further supports the critical role of mechanotransduction in maintaining tissue homeostasis [10, 11]. It has been reported that biomechanics and mechanical signaling mediate a variety of uterine physiological and pathological processes, including uterine leiomyoma, pregnancy and delivery [12, 13]. Although substantial research on other organs has confirmed that mechanical cues affect tissue repair after injury, few studies have focused on uterine tissues. Understanding the molecular mechanisms involved in the complex interplay between endometrial stromal cells (ESCs) and the surrounding ECM represents one of the major challenges in research on repair after endometrial injury.

Intriguing topics that have been addressed in recent studies in the field of regenerative medicine have included mechanochemical coupling and force sensing through the Hippo pathway [10, 14]. Hippo signaling is an evolutionarily conserved kinase cascade and a key pathway that regulates the size of mammalian organs [15]. The transcriptional regulators YAP and TAZ participate in core kinase cascades of the Hippo pathway and act as sensors and mediators of biochemical and mechanical cues that regulate cell proliferation and differentiation [16, 17]. Interestingly, YAP/TAZ activity appears to be dispensable for the development of some tissues (e.g., the intestinal epithelium and liver hepatocytes) but is critical in promoting tissue repair after injury. Numerous studies have revealed that YAP and TAZ are involved in the regeneration of organs such as liver, heart, and lung and in wound healing through somatic stem cell regulation, alterations in the microenvironment and responses to mechanical forces in damaged tissues [14, 18–20]. However, few studies have focused on whether YAP regulates repair after uterine injury or periodic regeneration during the menstrual cycle.

## Results

**Fig. 1:**
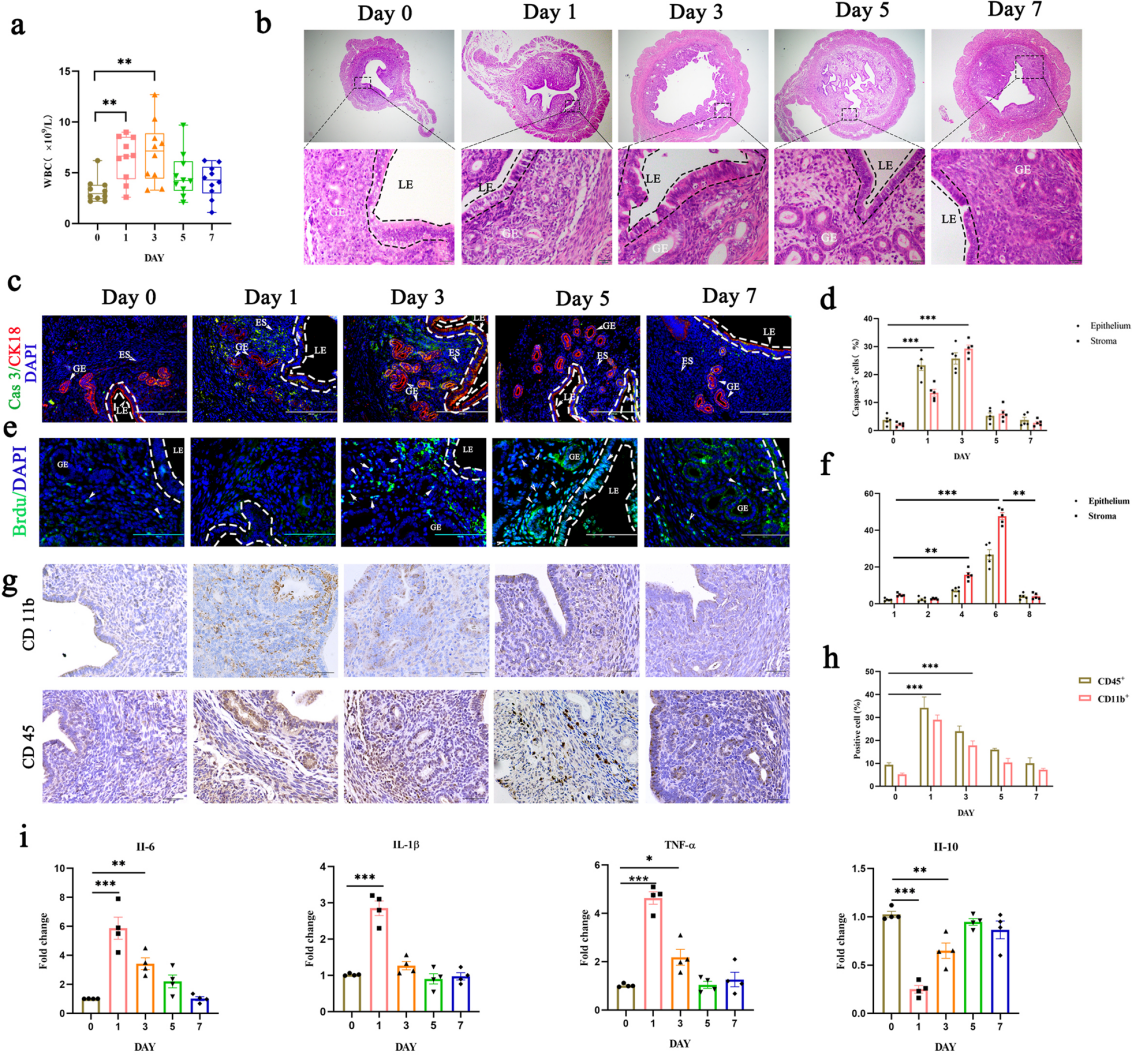
Endometrial injury and recovery in inflammatory-infected mouse uterus. Uterine tissues and blood were collected at 1, 2, 4, 6, and 8 d after injection of LPS. **a** White blood cell (WBC) content was analyzed by routine blood examination. n = 10–12 mice per group. **b** Representative histopathological images of paraffin-embedded mouse uterine sections. Scale bar: 200 μm (top); 20 μm (bottom). **c–f** Representative images (left panels) and quantification (right panels) of caspase 3 (**c**, **d**) and BrdU (**e**, **f**) expression in the indicated tissues from mice with inflammatory damage and in control mice. Scale bars, 200 μm. **g**, **h** Immunostaining of CD11b and CD45 (**g**) and quantification (**h**) of their staining in uterine tissue sections from mice with inflammatory damage. Scale bar: 20 μm. **i**. mRNA levels of IL-6, IL-1β, TNF-α and IL-10 in the endometrium were measured by RT‒qPCR. Data are means ± S.E.M. of at least three independent experiments. *P < 0.05; **P < 0.01; ***P < 0.001

To explore the mechanisms through which endometrial repair occurs, we used a pathological mouse uterine injury model generated by intrauterine injection of LPS. Although the LPS-induced mouse endometritis model has been widely recognized, most research using this model has focused on the damage phase, and the pathological characteristics of the repair phase have not been clearly explained [21, 22]. First, we evaluated the levels of white blood cells (WBCs) in the peripheral blood of mice with bacterially induced uterine injury by routine blood b examination (Fig. 1a and Additional file 1: Fig. S1b). Morphologically and histologically, the superficial GE and LE displayed obvious shedding after 24 h of LPS treatment, and they had fully recovered by Day 8 after reaching a peak on Day 4 (Fig. 1b–f, Additional file 1: Fig. S1c–e). As expected, we found that intrauterine injection of LPS led to massive apoptosis in the superficial GE and LE of the uterus and that the tissue then recovered concurrent with ESC proliferation (Fig. 1c–f). The process of endometrial injury is always accompanied by increased infiltration of the lamina propria by inflammatory cells. Immunostaining showed that the number of cells positive for CD45 or CD11b, markers of immune cells and macrophages, increased after LPS treatment but that these cells had almost disappeared by the repair phase (Fig. 1g, h). The level of secretion of inflammatory factors such as IL-6, TNF-α and IL-10 in the uterine tissue after injury was measured by qPCR, and the results were consistent with the above observations (Fig. 1i). We also used a repetitive injury model created by intrauterine injection of mouse LPS on Day 1, Day 8, and Day 15 (3 × LPS) to simulate prolonged chronic inflammatory damage in patients by bacteria. The corresponding experimental results showed that the damage induced by 3 × LPS is more obvious than that induced by a single injection of LPS; the uterine wall becomes thinner, but this has no obvious effect on the recovery period (Additional file 1: Fig. S1f–h).

The endometrium of postpartum mice provides a model of physiological mechanical injury. In this study, we found that the postpartum uterus initiated repair on Day 5 after delivery and that it had fully recovered to the nonpregnant state by Day 10 (Additional file 1: Fig. S1i and j). Of note, the period of repair of postpartum uterine injury is accompanied by a transition of the uterine environment from a proinflammatory state to an anti-inflammatory state (Additional file 1: Fig. S1k). The results described here indicate that endometrial regeneration occurs after the induction of uterine injury by physiological and pathological factors.

### Biomechanics of the regeneration of endometrial injury

**Fig. 2:**
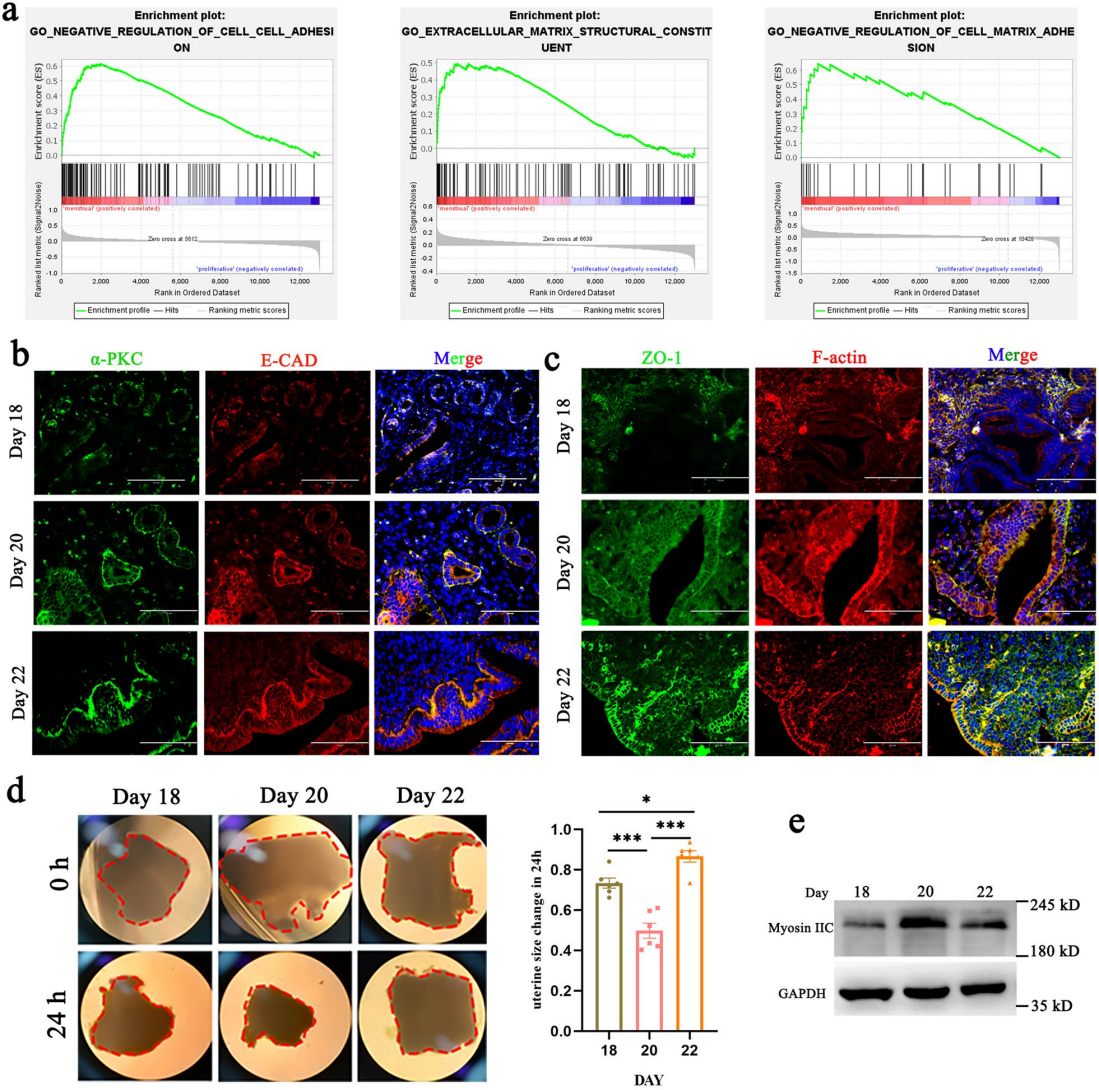
Changes in biomechanical cues during endometrial regeneration. **a** Gene set enrichment analysis (GSEA) of RNA-seq data for menstrual and proliferative cells revealed enrichment of cell‒cell adhesion gene signatures, ECM structural signaling-related genes, and cell matrix adhesion in proliferative cells. The normalized enrichment scores (NES) were 2.51, 1.95 and 2.10, respectively. The false discovery rates (FDR) were 0.000, 0.045 and 0.003, respectively. *P* < 0.001. **b**, **c** Immunofluorescence staining of α-PKC and E-cad (**b**) and F-actin labeled by phalloidin and ZO-1 (**c**) in sections of mouse uterus. Scale bar: 100 μm. **d** Uterine explants obtained at different repair phases at the beginning and 24 h later (left) and changes in uterine explant area over a period of 24 h (right). Scale bars, 1 mm. **e** Lysates of uterine tissue obtained at different repair phases were analyzed for the presence of the indicated proteins. Data are means ± S.E.M. of at least three independent experiments. *P < 0.05; ***P < 0.001

To gain insight into transcriptional regulation during repair after injury, we examined RNA-sequencing data about the damage and repair of human endometrium during the menstrual cycle obtained from a public database (GSE111976) [23]. GO term and gene set enrichment analysis (GSEA) revealed changes in cell‒cell adhesion, cell-ECM adhesion and ECM structural constituents (Fig. 2a). Because mechanical forces depend on focal adhesion and on ECM stiffness, we speculated that mechanical force drives the repair of the endometrium. To confirm our speculation, we first traced endometrial cell polarization by analyzing atypical protein kinase C (α-PKC) and E-cadherin (E-CAD) expression from Day 18 to Day 22. The results suggested that, after LPS treatment, the expression of E-CAD at the cell surface decreases and the apical-basal polarity of epithelial cells is lost. During the repair period, E-CAD and α-PKC redistributed to the apex and base of the cell (Fig. 2b). Because the establishment of correct apical–basal cell polarity is linked to the integrity of the cytoskeleton and to cell‒cell adhesion, we also stained uterine tissue sections for ZO-1 and actin filaments; the results suggested that cell adhesion and F-actin contractility decreased after injury and recovered during endometrial repair (Fig. 2c). In vitro, we cultured full-thickness uterine explants for 24 h and quantified the uterine area ratio at 0 h and 24 h. The results suggested that there is a notable decrease in this ratio on Day 20 compared to Day 18 and Day 22 (Fig. 2d). Western blotting for myosin IIC, which binds to F-actin and thereby generates contractile force, showed that there is an increase in the level of this protein during the repair period (Fig. 2e). We also observed this phenomenon during the process of postpartum uterine involution in mice (Additional file 1: Fig. S2a–c), and it is consistent with previous transcriptomics data (GSE40312) [24] for the postpartum endometrium of dairy cows (Additional file 1: Fig. S2d). Although species differ, this observation appears to suggest a general phenomenon associated with mammalian uterine repair. The data suggest that mechanical cues are involved in regulating repair after uterine injury.

### YAP activation drives endometrial regeneration

**Fig. 3:**
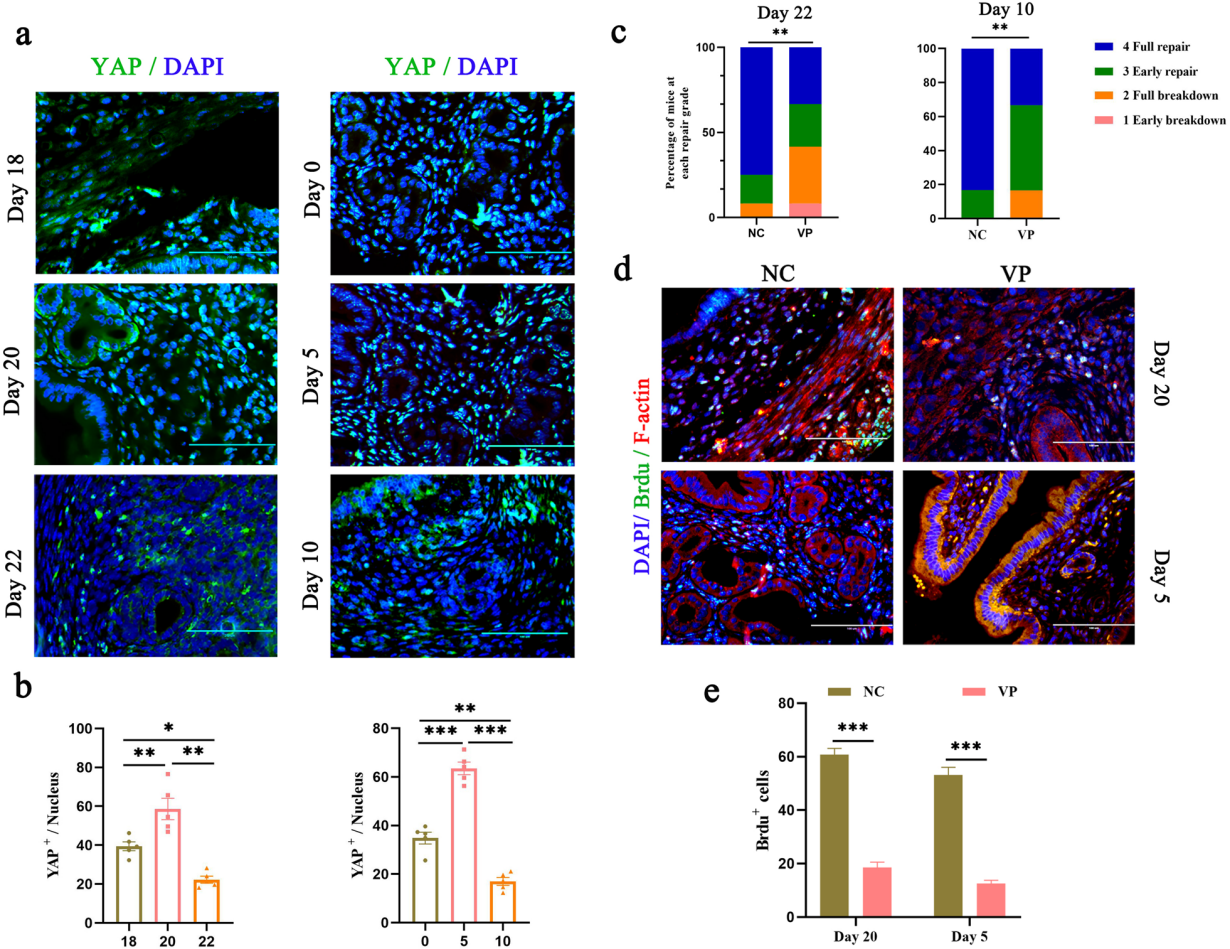
Expression and function of YAP in mouse uterus during the repair phase. **a**, **b** Inflammatory-infected and postpartum mouse uterine sections stained with antibodies against YAP (**a**) were examined by immunofluorescence microscopy, and quantification of YAP+ positive cells was performed (**b**). Scale bars, 100 μm. **c** Percentage of sections encompassing each of morphological stages for the negative control (NC, DMSO) and verteporfin (VP) groups on Day 22 and Day 10. **d**, **e** Immunofluorescent images of uterine sections from inflammatory-infected mice stained with antibodies against BrdU (**d**) at Day 20, postpartum mice at Day 5, and quantification of BrdU+ positive cells (**e**). Scale bars, 100 μm. The data are presented as mean ± S.E.M. Data are means ± S.E.M. of at least three independent experiments.. *P < 0.05; **P < 0.01; ***P < 0.001

As we demonstrated above, the dynamic biomechanical nature of endometrial remodeling and the endometrial cell regeneration that occur during the repair of physiological or pathological uterine lesions are remarkable, but the mechanism by which cells in the endometrial cellular niche respond to mechanical cues remains unclear. We previously showed that YAP, as a sensor of chemical and mechanical cues, participates in the establishment of pregnancy [25]. Therefore, we speculate that YAP is involved in the conversion of mechanical signals to chemical cues during the uterine repair process. As expected, on Day 20 after mice were treated with 3 × LPS or on Day 5 after delivery, the level of YAP protein was increased, and the protein was translocated into the nucleus, indicating that the activity of YAP mediates endometrial repair (Fig. 3a, b and Additional file 1: Fig. S3a). Expression of YAP and of its target genes CTGF, CYR61, and ANKRD1 was high during the uterine repair process (Additional file 1: Fig. S3b, c).

Verteporfin (VP), an FDA-approved compound that is used in the treatment of macular degeneration, blocks the interaction between YAP and TEAD and disrupts YAP nuclear activity [14, 26]. To verify the importance of YAP activation in endometrial regeneration after LPS-induced damage, we blocked YAP-TEAD interactions during LPS exposure. Inhibition of YAP extended the repair time, as shown by the decreased scores of uterine sections (Fig. 3c), and obviously inhibited endometrial cell proliferation (Fig. 3d, e). Consistent with this, mice that were directly intraperitoneally injected with siYAP showed a notable increase in repair time (Additional file 1: Fig. S3d, e). Taken together, these findings identify YAP activation as a dominant driver of endometrial regeneration following damage.

### YAP is regulated by ECM stiffness in ESCs

**Fig. 4:**
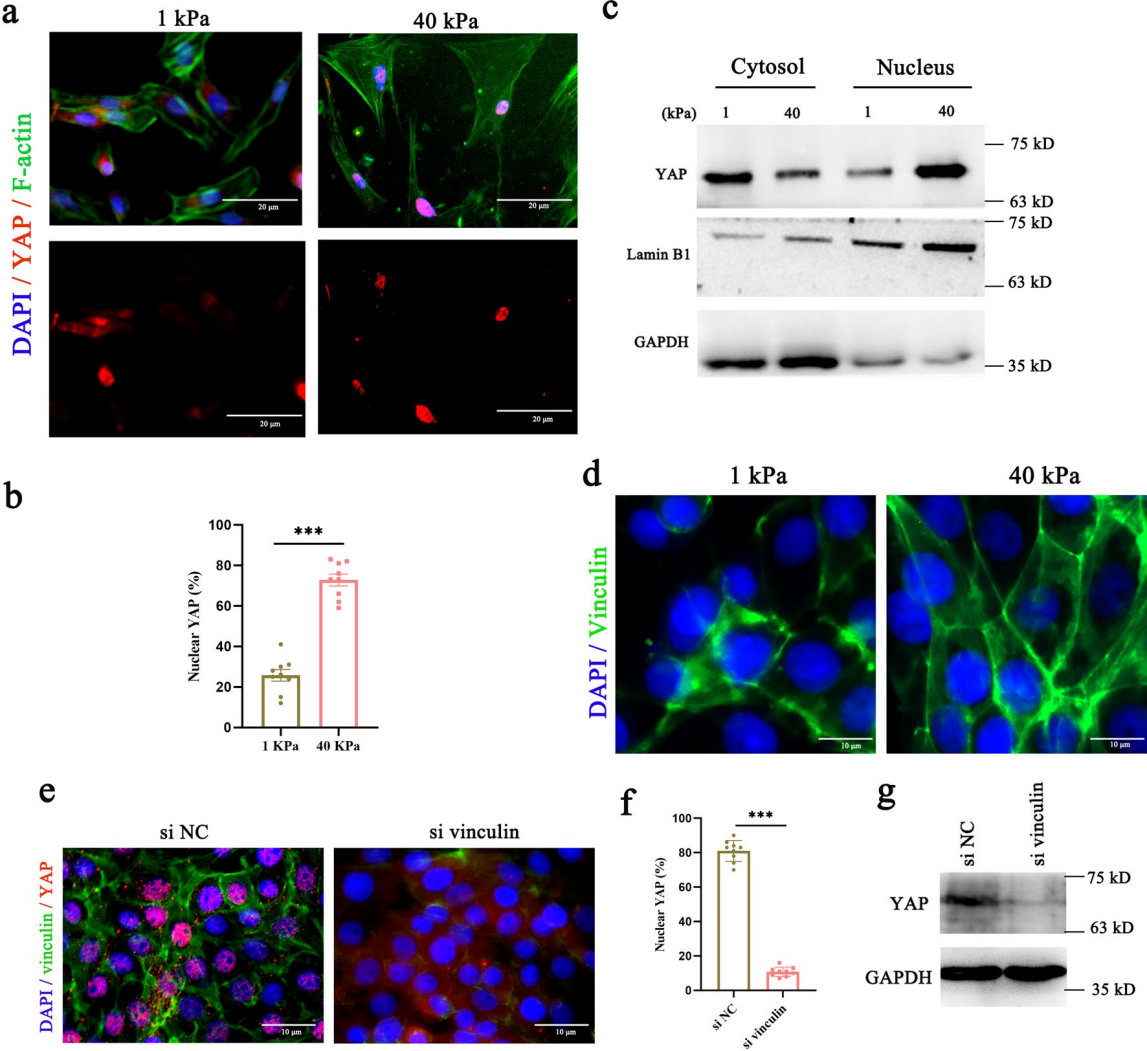
YAP responds to mechanical signaling via focal adhesion in ESCs. **a**, **b** Confocal immunofluorescence images (**a**) and quantification of nuclear and cytoplasmic subcellular localization (**b**) of YAP in ESCs plated on hydrogels with different rigidities. Scale bars, 20 μm. **c** Western blotting for YAP in nuclear and cytoplasmic protein fractions from ESCs plated on 1 kPa or 40 kPa fibronectin-coated hydrogels for 24 h. **d** Representative immunofluorescence images of vinculin in ESCs cultured on 1 kPa and 40 kPa hydrogels, n = 2. Scale bars, 10 μm. **e**, **f** Immunofluorescence images of YAP and vinculin (**e**) and quantification of YAP+ positive cells (**f**) in ESCs. Scale bar: 10 μm. **g** Lysates of ESCs transfected with si vinculin were analyzed for the presence of the indicated protein. Data are means ± S.E.M. of at least three independent experiments. ***P < 0.001

Structurally and functionally, menstruation and pathological factors cause shedding of the functional layer of the uterus, while the basalis layer remains intact and is available for uterine repair [27, 28]. Therefore, we isolated ESCs to explore crosstalk between YAP and mechanical cues during endometrial repair. The ESC marker vimentin was used to determine the purity of the isolated ESCs (Additional file 1: Fig. S4a). ECM stiffness is the most important mechanical signal in maintaining the homeostasis of the extracellular microenvironment. We found that ESCs grown in stiff hydrogels (~ 40 kPa) induce YAP activation compared to ESCs grown in soft matrices (~ 1 kPa) (Fig. 4a–c, Additional file 1: Fig. S4b). Adherens junctions are mechanosensitive, responsive structures that link the cytoskeleton to the ECM [29]. After staining of ESCs for vinculin, a marker of adherens junctions, we observed that vinculin expression decreased in ESCs grown in soft matrices (Fig. 4d). A previous study suggested that vinculin interacts with TRIP6 in response to mechanical forces induced by cell density [30]. Here, we observed that knockdown of vinculin prevented the YAP activation that was otherwise induced by ECM stiffness (Fig. 4e–g, Additional file 1: Fig. S4c). These data indicate that the regulation of YAP activity by ECM stiffness depends on focal adherence.

### Rap1a interacts with p190A to mediate the regulation of YAP by ECM stiffness

**Fig. 5:**
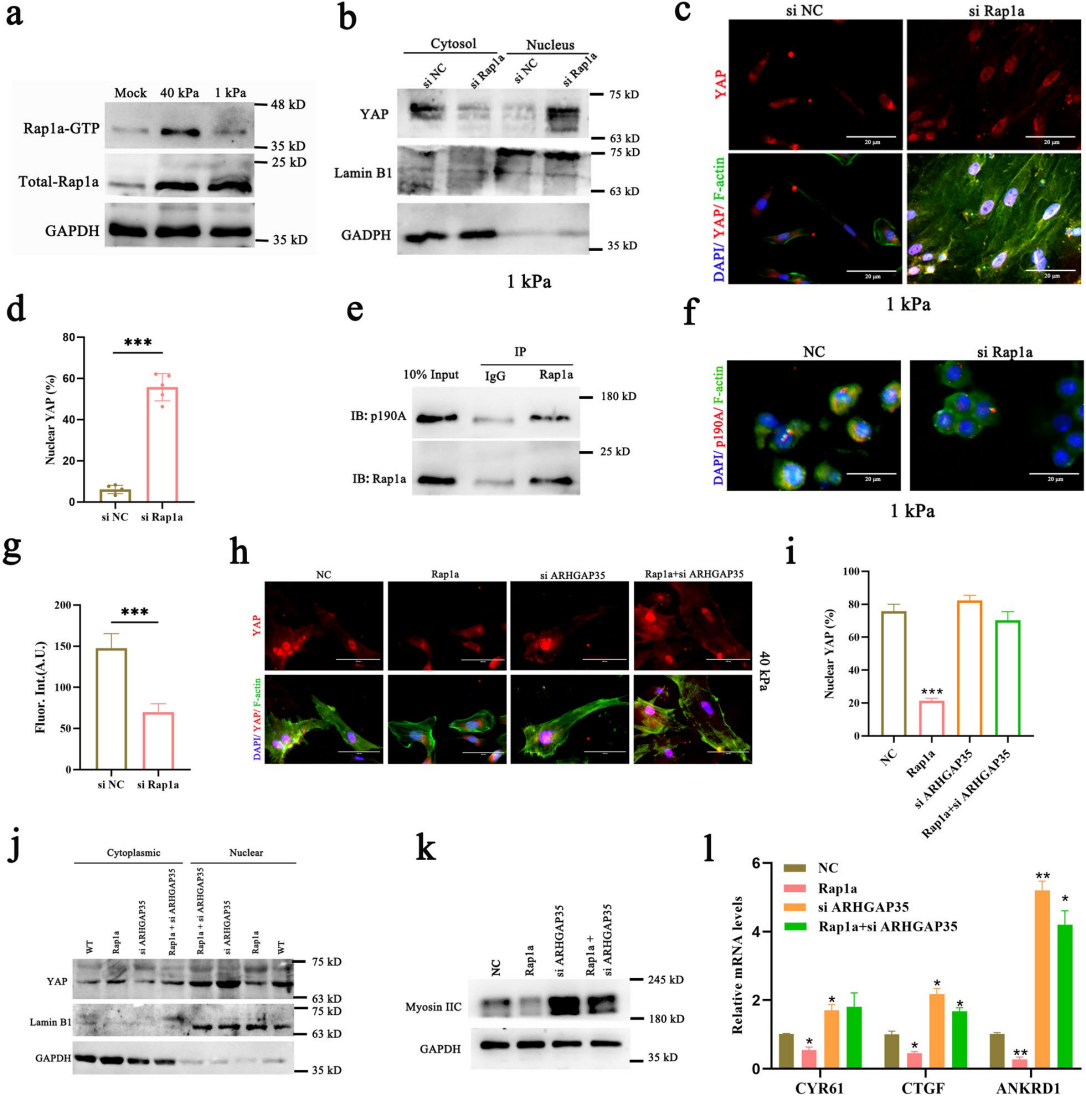
ECM stiffness acts via RAP1a/ARHGAP35 to regulate YAP in ESCs. **a** Pull-down assays of total Rap1a and activated Rap1a (Rap1a-GTP) in ESCs plated on 1 kPa and 40 kPa hydrogels for 24 h. **b** Western blotting for YAP in nuclear and cytoplasmic protein fractions from ESCs transfected with si Rap1a and plated on 1 kPa fibronectin-coated hydrogels for 24 h. **c**, **d** Confocal immunofluorescence images (**c**) and quantification of the nuclear and cytoplasmic subcellular localization (**d**) of YAP in ESCs plated on 1 kPa hydrogels and transfected with si Rap1a. Scale bar: 20 μm. **e** Coimmunoprecipitation (IP) of p190A and Rap1a in ESCs plated on 1 kPa hydrogels. Cell lysates were immunoprecipitated with Rap1a antibodies or with the corresponding IgG controls and probed with the indicated antibodies. **f**, **g** Confocal immunofluorescence images (**f**) and quantification of the average fluorescence intensity (**g**) of p190A in ESCs. Cells were plated on 1 kPa fibronectin-coated hydrogels and transfected with si Rap1a or si NC for 24 h. Scale bar: 10 μm. **h–j**. ESCs were plated on 40 kPa fibronectin-coated hydrogels and transfected with pcDNA3.1+ Rap1a or si ARHGAP35 or pcDNA3.1+ Rap1a and si ARHGAP35 or pcDNA3.1+ NC for 24 h. **h**, **i.** Confocal immunofluorescence images (**h**) and quantification of the nuclear and cytoplasmic subcellular localization (**i**) of YAP in ESCs. Scale bar: 10 μm. **j**, **k** Total cell lysates or nuclear and cytoplasmic lysates of ESCs were prepared and subjected to immunoblot analysis with the indicated antibodies. **l** RT‒qPCR analysis of CYR61, CTGF and ANKRD1 mRNA levels in ESCs. Data are means ± S.E.M. of at least three independent experiments. *P < 0.05; **P < 0.01; ***P < 0.001

In considering the mechanism through which ECM stiffness signals YAP, we speculate that small GTPases, which function as molecular switches in many biological processes, may affect the localization of YAP in ESCs seeded on ECM of different stiffnesses. Here, we found that the activity of Rap1a was significantly attenuated in ESCs grown on stiff hydrogels (Fig. 5a). Rap1a was identified because its knockdown induced nuclear translocation of YAP even in cells seeded on a soft matrix (Fig. 5b–d, Additional file 1: Fig. S5a). Consistent with this finding, overexpression of Rap1a eliminated YAP activation induced by high ECM stiffness (Additional file 1: Fig. S5b–d). It should be noted that other GTPases of the Rap1 family do not have similar activity (Additional file 1: Fig. S5e). The expression of CTGF, CYR61, and ANKRD1 was repressed in wild-type ESCs seeded on ECM of low stiffness, but this did not occur in the RAP1 knockdown group (Additional file 1: Fig. S5f). Rho GTPases coordinate changes in cytoskeletal architecture, are controlled by RhoGEFs and RhoGAPs [31]. Interestingly, we noted that p190A, which is encoded by the ARHGAP35 gene and is a member of the Rho GAP family, was interacts with Rap1a in ESCs grown on ECM of low stiffness (Fig. 5e). By staining for p190A in ESCs grown on 1 kPa hydrogel, we found that knockdown of Rap1a significantly inhibited its expression (Fig. 5f, g). We speculated that p190A is a downstream effector protein of Rap1a and that it mediates mechanotransduction. Knockdown of p190A induced actin polymerization and stress fiber formation, which can antagonize the inhibition of YAP activity caused by overexpression of Rap1a in cells grown on 40 kPa hydrogel (Fig. 5h–k, Additional file 1: Fig. S5g). This result was also reflected in the levels of expression of downstream target genes of YAP (Fig. 5l). In ESCs grown on 1 kPa hydrogel, we also found that the YAP inhibition caused by low ECM stiffness was improved after knockdown of p190A (Additional file 1: Fig. S5h, i). These results suggest that the complex of Rap1a and p190A acts as a molecular switch that mediates mechanotransduction following changes in local adhesion.

### Rap1/ARHGAP35 inhibits RHOA through ARHGAP35

**Fig. 6:**
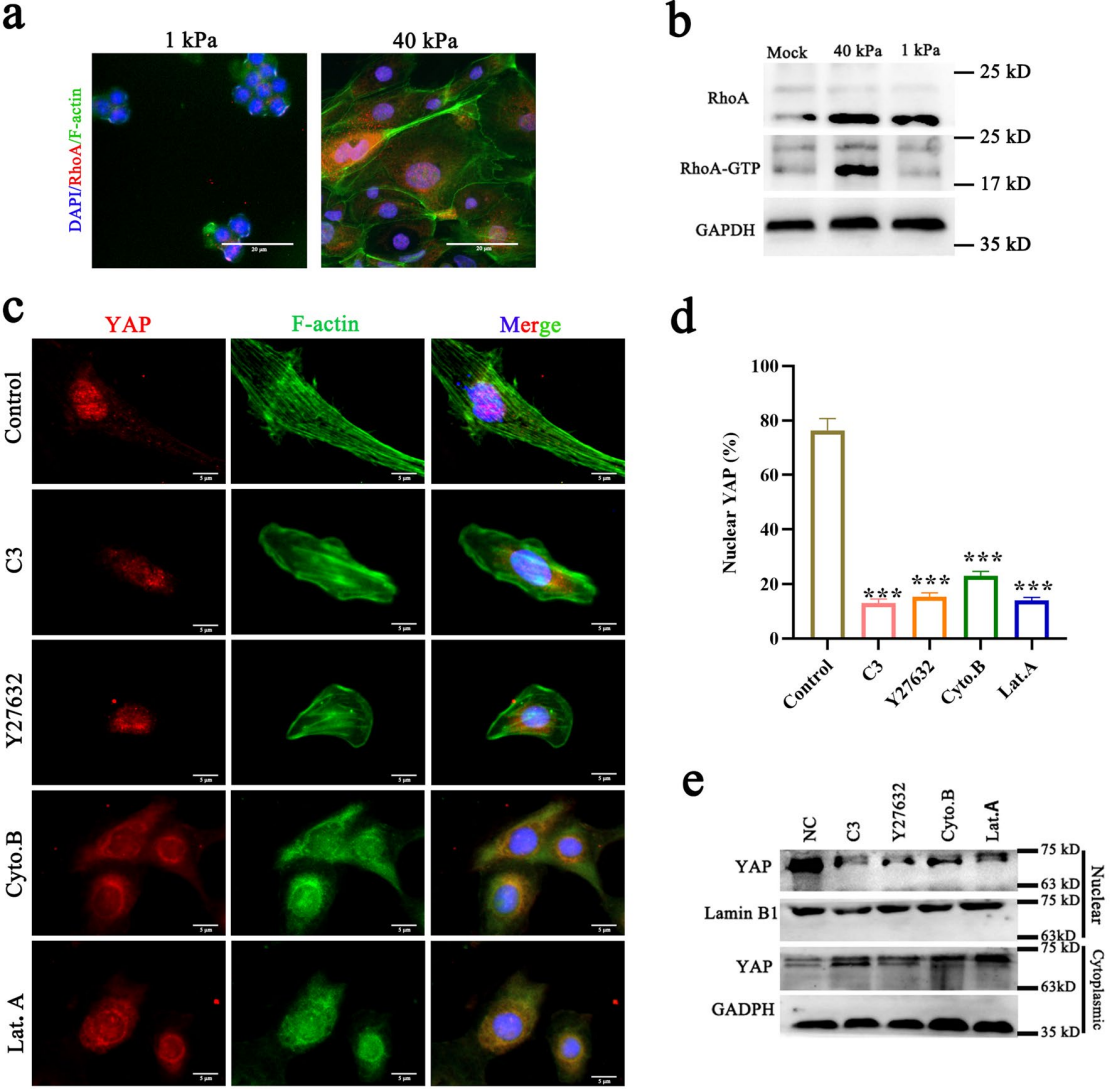
ECM stiffness regulates YAP activity through the Rap1a/ARHGAP35/RhoA/F-actin pathway. **a** Immunofluorescence staining showing the expression of RhoA in ESCs. **b** Pull-down assays of total RhoA and activated RhoA (RHOA-GTP) in ESCs plated on 1 kPa and 40 kPa hydrogels for 24 h. Scale bars, 20 μm. **c**, **d** Confocal immunofluorescence images (**c**) and quantification of the nuclear and cytoplasmic subcellular localization of YAP (**d**) in ESCs. Cells were plated on 40 kPa fibronectin-coated hydrogels and treated with C3, Y27632, or Lat. A and Cyto. B for 24 h. Scale bars, 5 μm. **e** Lysates of ESCs treated with C3, Y27632, or Lat. A and Cyto. B for 24 h were analyzed for the presence of the indicated proteins. Data are means ± S.E.M. of at least three independent experiments. ***P < 0.001

Rho GTPases are critical regulators of the cytoskeletal architecture that coordinate fundamental cell behaviors in eukaryotic cells [32]. A previous study reported that ARHGAP35 exhibits inhibitory activity toward RhoA but not toward Rac1 or CDC42 [31]. Therefore, we evaluated RhoA activity by immunofluorescence in ESCs that were in a highly contractile state. We discovered that the activity of RhoA was obviously increased in ESCs on hydrogels with 40 kPa stiffness compared to ESCs on hydrogels with 1 kPa stiffness; this implies that RhoA is involved in the transduction of signals related to ECM stiffness (Fig. 6a, b). In this study, we evaluated the activation of YAP after inhibition of RhoA activity by treatment of cells with C3 and Y27632, which are RhoA and ROCK inhibitors. The results indicated that both inhibitors significantly inhibited YAP activation (Fig. 6c, d). We also observed that the actin cytoskeleton was involved in mechanotransduction in the above scenario. We then treated ESCs with the F-actin inhibitors cytochalasin B (Cyto. B) and latrunculin A (Lat. A). We found that inhibition of F-actin polymerization interfered with YAP activation (Fig. 6c, d). The above results were also verified in experiments that involved the separation of nucleoplasmic proteins (Fig. 6e). These results reveal the existence of a signaling axis, the focal adhesion/Rap1a/ARHGAP35/RhoA/F-actin/YAP axis, that links ECM stiffness with mechanotransduction.

### Mechanotransduction aids uterine regeneration by inducing ESC proliferation in vitro and in vivo

**Fig. 7:**
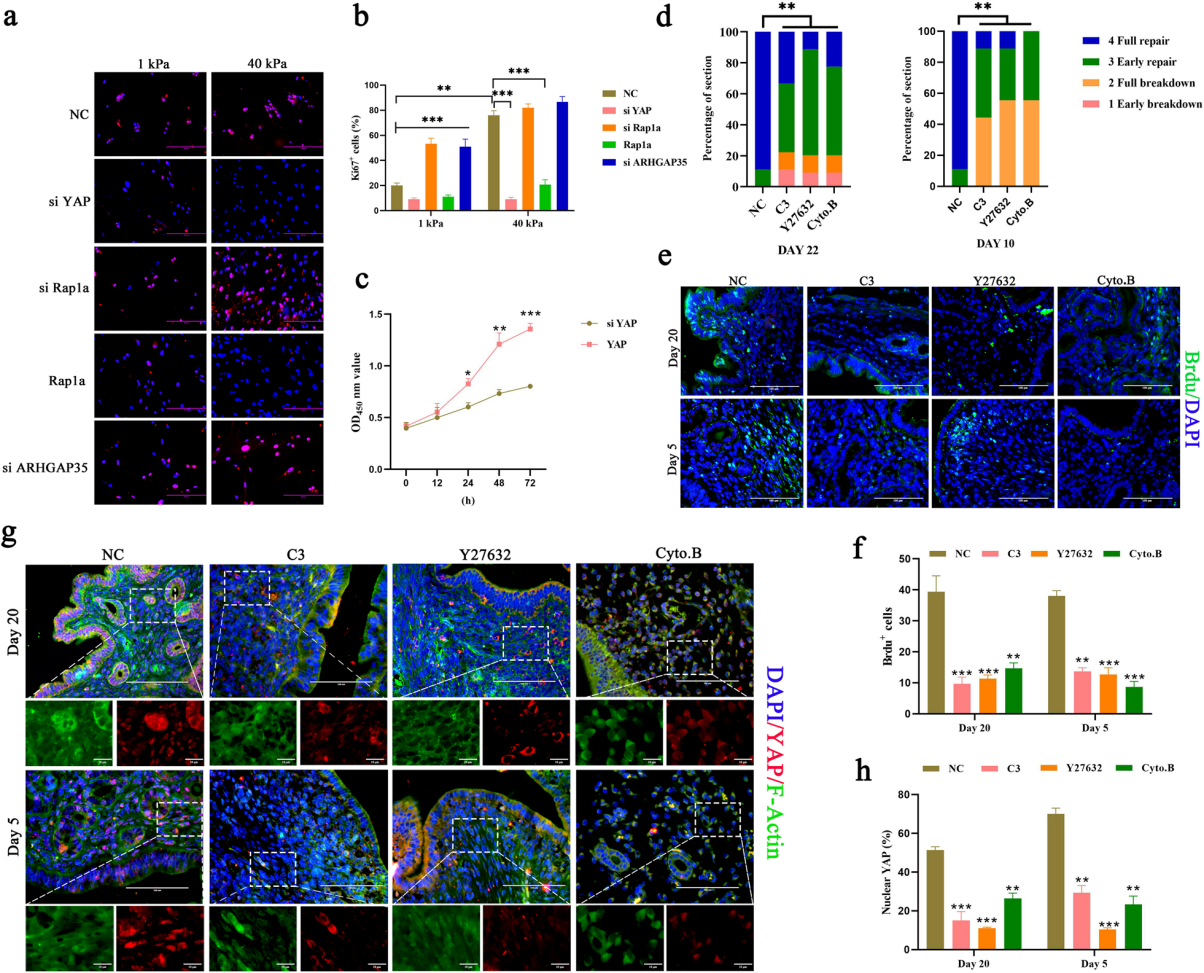
Mechanotransduction contributes to ESC proliferation during uterine regeneration in a Rap1a/ARHGAP35/RhoA/F-actin YAP-dependent manner. **a**, **b** Immunofluorescence images (**a**) and quantification of Ki67-positive cells (**b**) in ESC cultures transfected with si NC, si YAP, si Rap1a or pcDNA3.1(+) Rap1a at 40 kPa/1 kPa hydrogels. Scale bars, 200 μm. **c** CCK-8 kits were used to assess the proliferation of ESCs transfected with siYAP or pcDNA3.1(+) YAP at 0, 12, 24, 48 and 72 h. **d–h** Uterine tissues were collected from inflammatory-infected and postpartum mice treated with C3, Y27632 and Cyto. B on Day 20, Day 5, Day 22 or Day 10. **d** Percentage of sections encompassing individual morphological stages at Day 22 or Day 10. **e**, **f** Confocal immunofluorescence images (**e**) and quantification of BrdU+ positive cells (**f**) in uterine sections. Scale bar: 100 μm. **g**, **h** Confocal immunofluorescence images (**g**) and quantification of the nuclear and cytoplasmic subcellular localization of YAP (**h**). Scale bar: 100 μm, 10 μm (bottom). Data are means ± S.E.M. of at least three independent experiments. *P < 0.05; **P < 0.01; ***P < 0.001

Next, we found that knockdown of Rap1a or ARHGAP35 markedly increased ESC proliferation and that overexpression of Rap1a abolished proliferation induced by high ECM stiffness (Fig. 7a, b). Consistent with our previous study, in which wer reported that YAP promotes the proliferation of EECs [25], we found that the proliferation of ESCs is also affected by YAP activation (Fig. 7c).

To verify the pathway of mechanotransduction that we observed in vitro, we used inhibitors of critical proteins in this pathway to interfere with mechanical conduction in an injured mouse model. We administered C3, Y27632 and cytochalasin B, which are ROCK- and F-actin-specific antagonists, respectively, to the uteri of mice in the initial repair phase. The results suggest that administration of C3, Y27632 and cytochalasin B prolongs the repair time of the uterus (Fig. 7d, Additional file 1: Fig. S6a, b) and inhibits cell proliferation (Fig. 7e, f, Additional file 1: Fig. S6c, d). As we speculated, the results of immunoblotting and immunofluorescence staining showed that these inhibitors attenuated YAP activation (Fig. 7g, h, Additional file 1: Fig. S6e). Compared with the control group, the treatment of these inhibitors had no significant effect on the level of phospho-YAP (Additional file 1: Fig. S6e). Of note, cell proliferation was not completely suppressed, and it remains unclear whether this was due to incomplete inhibition or whether other cues that are independent of F-actin are involved in the repair of the endometrium. For example, the classical Hippo-YAP pathway is regulated by chemical signals, and the WNT signaling pathway regulates stem cell activation and proliferation[25, 33, 34]. Together, these data indicate that blocking the mechanotransduction pathway leads to prolongation of the time required for uterine repair.

## Discussion

**Fig. 8:**
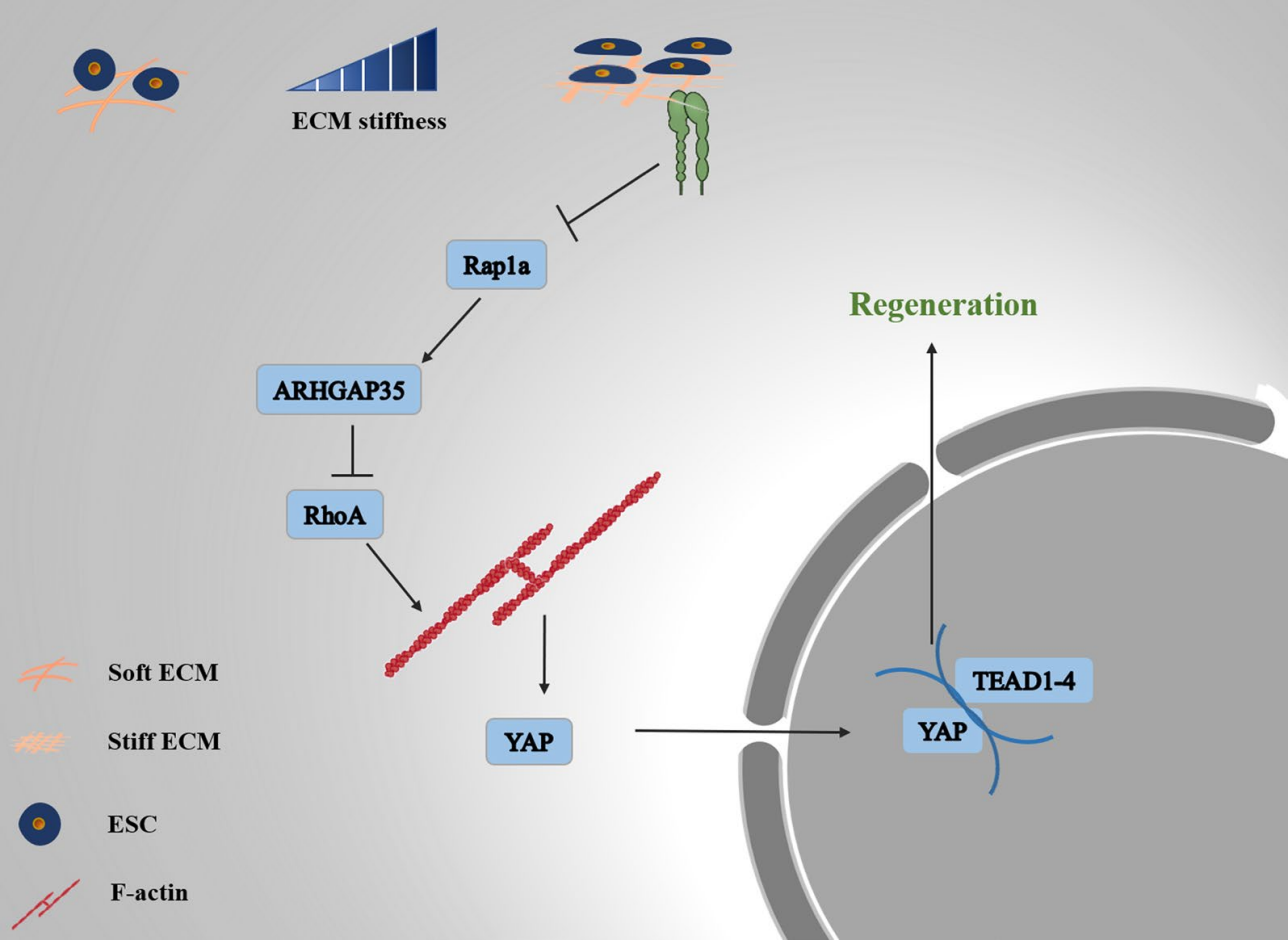
Schematic model of mechanotransduction in endometrium repair

To cope with organ injury, a complex mechanism for achieving functional regeneration exists in mammalian cells. Timely, scarless repair after physiological or pathological injury of the uterus is a key factor in maintaining the fertility of female animals [35, 36]. Mechanical forces have a long-established role in regulating cell fate and behavior through processes of mechanotransduction, and it is increasingly recognized that such forces also have other roles such as controlling proliferation, apoptosis and cellular metabolism [6, 7, 37]. Understanding how mechanical forces spatiotemporally orchestrate regeneration processes is a long-standing challenge. A central question is how mechanical cues are transduced in cells when the uterus is damaged. In this study, we found that dynamic mechanical forces drive regeneration after uterine damage and that the mechanical force is transmitted through the focal adhesion/Rap1a/ARHGAP35/Rho A/F-actin/YAP axis (Fig. 8).

In this work, we first define the repair period after uterine injury, especially the repair period for biological injury and mechanical injury, by evaluating the dynamic characteristics of the endometrium. Of note, the amount of time required to repair the uterus is related to the type of animal and to various factors related to individual differences. This study explores a general condition, and the completion of the repair is not based on the body's re-estrus. Multiple cell lineage tracking studies indicated that the completion of endometrial repair mainly occurs due to the proliferation and differentiation of progenitor cells in the basal layer [27]. The results obtained in this study on the proliferation and apoptosis of ESCs and EECs during the repair period also illustrate this phenomenon. These results provide evidence that supports the selection of ESCs in the next in vitro study.

The endometrium is an organ that is sensitive to mechanical force and to mechanical cues that regulate internal tension and external force. Our previous study and other data suggested that mechanotransduction is involved in the physiological and pathological processes that occur in the uterus, including embryo implantation, the development of uterine fibroids, and childbirth [25, 38]. We now show that uterine injury results in a loss of EEC polarity and in breakage of TJs and AJs between cells and that GO terms related to mechanotransduction and mechanical force formation are activated and are accompanied by actin polymerization and high expression during the repair phase. Relevant studies have shown that mechanical force stimulation at the level of tissues and organs can improve the prognosis of injury and disease, including injuries and diseases of the skin, the musculoskeletal system and nerves [39, 40], but the mechanism of this effect is not clear. Increasing evidence shows that YAP, as a sensor and mediator of mechanical cues, participates in a variety of physiological and pathological processes that are mediated by mechanical forces, including repair and regeneration after tissue injury [14, 41, 42]. For example, after surgical removal of part of the lung, CDC42 transmits mechanical tension to activate YAP/TAZ and thereby participates in lung repair [43]. The response of YAP to mechanical force in damaged tissues is one of the classic themes in the field of regenerative medicine research. In this study, we found that YAP was activated during the repair phase of the uterus and that it was inactivated after uterine repair was completed. After inhibition of the Hippo-YAP signaling pathway or direct knockdown of YAP expression in mice, the time required to repair the endometrium was significantly prolonged.

Research on the relationship between mechanical cues and tissue repair focuses on elucidating the function of cell‒cell and cell‒ECM interactions, both of which participate in the generation of internal mechanical forces and mechanotransduction. Here, we found that ECM stiffness regulated the activity of YAP by dependent focal adhesion in ESCs. Of note, the response to mechanical cues has specificity for the type of tissue and for cell type, due to the spatial location of the tissue and the threshold that the cell can withstand [44]. As a GTPase, Rap1 is normally in the active GTP-bound form; it is the main regulator of actin dynamics and is very important in the formation of cell movement connections [45, 46]. Moreover, Rap1 is also a sensor of ECM stiffness [47]. We showed in this study that the response of YAP to ECM stiffness and cell adhesion is blocked by Rap1a in a manner that directly leads to changes in YAP expression and subcellular localization. Rap1a also induces F-actin aggregation, indicating that the ability of a single ESC to sense and respond to ECM stiffness depends on the presence of Rap1a. A previous study showed that activation of Rap1 is involved in the establishment of cell polarity [47]. Our results indicate that epithelial cell polarity is rebuilt during uterine repair, suggesting that Rap1a is involved in the repair of endometrial injury.

Our data suggest that the induction of cytoplasmic metastasis by YAP is blocked by low ECM stiffness or by Rap1a activation through reduction of the expression of ARHGAP35. Combined with the observed interaction between Rap1a and ARHGAP35 in ESCs cultured on ECM of low stiffness, these results show that ARHGAP35 is a component of a core protein mechanotransduction pathway. Substantial research indicates that ARHGAP35 is a member of a group of GTPase-activating proteins (RhoGAPs); it encodes p190A RhoGAP (p190A), the functions of which are related to cell adhesion, cell migration, gene transcription and protein translation [48–50]. It should be noted that inactivation of ARHGAP35 leads to abnormal activation of RhoA in endometrial cancer and promotes the malignant transformation of tumor cells through the Hippo-Yap pathway [51], consistent with the conclusions reported in this study. The RhoGAP and RhoGEF families primarily regulate the function of Rho GTPases [31, 52]. Rho GTPases coordinate changes in the structure of the cytoskeleton and in this way regulate basic cellular behavior in multicellular organisms, and interruption of Rho signaling is related to cancer metastasis and to many other diseases [31]. In further exploration of the mechanotransduction mechanism, we found that the activity of RhoA, a member of the Rho GTPase family, is inhibited by ARHGAP35 and that it participates in the transduction of mechanical cues in ESCs. Moreover, when the RhoA inhibitor Y27632 was used in the mouse model, uterine repair time was significantly prolonged. We suggest that F-actin participates in the reconstruction that occurs during the repair phase and that its polymerization directly promotes nuclear entry and high expression of YAP.

A previous study reported that Rap2a, but not Rap1b, H-RAS, K-RAS, or N-RAS, which are other members of the GTPase family, participate in the regulation of YAP activation by ECM stiffness through the ARHGAP29/RHOA/Hippo pathway [53]. The seemingly contradictory conclusion reached in this study does not exclude the impact of Rap1a, and there are differences in the choice of in vitro carriers, which are one of the key factors affecting mechanotransduction. The contribution of this possible serial mechanism in vivo to mechanotransduction and the choice of parallel mechanisms will be the subject of our future study.

Collectively, the reported observations suggest that the contractility of ESCs and mechanotransduction drive uterine repair by promoting cell proliferation. Mechanistically, our results reveal that endometrial cells transmit mechanical cues through the Rap1a/ARHGAP35/RhoA/F-actin/YAP pathway during the uterine repair phase. The discovery of this novel mechanism provides forward-looking and in-depth insight into mechanotransduction and brings hope that the application of mechanical force can be used in the field of regenerative medicine to promote the restoration of tissue structure and function.

## Materials and methods

### Reagents and plasmids

Verteporfin (VP) was from Macklin Biochemical (Shanghai, China). Latrunculin A (Lat. A) was from Santa Cruz Biotechnology. Cytochalasin. B, C3(Rho inhibitor) and Y27632 purchased from AbMole (USA). The overexpression plasmid pcDNA3.1(+) YAP and pcDNA3.1(+) Rap1a, and si Rap1a/b, si Rap2a/b/c, si YAP, and si ARHGAP35 were generated by GeneCreate (Wuhan, China).

### Primary culture

Mouse endometrial stromal cells (ESCs) were isolated and cultured as previously described [25]. In brief, uterine horns were harvested from mice and finely chopped using scalpel blades. Minced tissue was then incubated in 20–25 mL of DPBS (HyClone) sterile digestive solution, which contained 1% collagenase II (Sigma-Aldrich) at 37 °C for 1 h. Chelation buffer containing tissue was filtered through 40 μm filter mesh to remove undigested material, and centrifuged at 1000 r/min for 5 min, and after 3 additional washes in washing medium. The suspension was centrifuged at the cells were resuspended in DMEM/F-12 culture medium containing 15% FBS (Gibco), 50 IU/mL penicillin, 50 IU/mL of streptomycin, and 2.5 μg/mL of amphotericin B (all from HyClone). The cells were cultured in 6 or 12-well plates (Corning) or 25- or 75-cm^2^ flasks at 37 °C. The endometrial epithelial and stromal cell populations were isolated by differential adhesion to cell culture flasks. Primary cells were used in the next experiment after four passages.

### Cell culture, transfections and treatments

2D culture on hydrogels with high (40 kPa) or low (1 kPa) stiffness was performed as described elsewhere [25]. In brief, 10 μg/mL bovine fibrinogen (Solarbio, Beijing, China) was used to coat hydrogels activated with Sulfo-SANPAH (Thermo Fisher Scientific, USA) according to the needs of the cells.

ESCs at 60% confluence were exposed to 0.5 μM Lat. A, 0.5 μM Y27632, 0.5 μM Cyto. B or 3 μg/mL C3, and an equal volume of PBS for 24 h and were then collected and stored at – 20 ℃ for use in the next experiment. Transient transfection of ESCs was conducted using Lipofectamine 2000 (Invitrogen, CA, USA) as recommended by the manufacturer. Briefly, Lipofectamine 2000 and plasmid, siRNA and overexpression plasmids were separately dissolved in 500 μL of Opti-MEM I medium (Gibco), and the solutions were then mixed and incubated for 20 min at room temperature to form complexes. The prepared mixture was added to exponentially growing cells (4 × 105) seeded in 6-well plates for 12 h. All RNAi oligonucleotides and plasmids were obtained from GeneCreate (Wuhan, China). All transfections were performed in triplicate, and the cells were cultured in OPTI-MEM medium for 24 h for RNA studies or for 48 h for protein studies.

### Mice and experimental processing

Female (aged 6–8 weeks) and male (aged 8–10 weeks) Kunming (KM) mice were purchased from the Laboratory Animal Service Centre of Huazhong Agricultural University. All mouse experiments were conducted in accordance with the university guidelines on animal experimentation (HZAUMO-2015-12), and approval by the Animal Ethics Committee of Huazhong Agricultural University was obtained for all related procedures. The mice were maintained at room temperature with a 12-h dark–light cycles and free access to food and water.

To create the uterine inflammatory injury model, mice were given intrauterine injections of 30 μL lipopolysaccharide (LPS, 0.5 mg/kg, Sigma). Uterine tissue was collected from the LPS-treated mice on Days 1, 2, 4, 6, and 8. At least 10 mouse uteri were collected at each time point. The injured mice were randomly divided into groups of 7 as follows: control group (PBS, i.p.); verteporfin (VP) group, intraperitoneal injection of VP (10 μmol/kg) daily; si YAP and si NC group, intrauterine injection of siRNA (30 pmol) every other day; C3 group, injection of C3 (50 μg/kg) into the tail vein daily; Y-27632 group, intraperitoneal injection of Y-27632 (10 mg/kg) in the morning and evening, 24 h before uterine collection; and cytochalasin B group, intraperitoneal injection of cytochalasin B (50 mg/kg) 24 h before uterine collection (Additional file 1: Fig. S1a). n = 10–12 mice per group. The same amount of PBS was injected into control mice. All treatments were performed 72 h after LPS treatment unless otherwise stated in the Results.

The mouse postpartum uterus is a natural model of mechanical injury. Mouse uteri were collected on Days 1, 5 and 10 after delivery.

All mice received an intraperitoneal injection of 100 μL bromodeoxyuridine (BrdU, 75 mg/kg, Sigma‒Aldrich) 12 h prior to culling. Animals in which the experimental model failed were excluded from the study. Mice with dermatitis, urethritis or fight wounds were excluded from the experimental analysis.

### Uterus explant culture

Culture of uterine explants was used to evaluate tissue contractility with reference to previous related studies [39]. Removal of the mouse uterus was performed under sterile conditions, and the uterus was rinsed and placed in sterile PBS. The horn of the middle uterus was cut off longitudinally and placed on an agarose pad (4% agarose in sterile water mixed 1:1 with 10% FBS in DMEM (GIBCO), with doxycycline (2 mg/mL), and 1:100 penicillin/streptomycin (GIBCO)). Uterine explants on agarose were gently placed in a 6-cm dishes containing 1 mL 10% FBS in GIBCO-DMEM (to avoid drying of the agarose) and cultured at 37 ℃ in 7.5% CO2 for at least 2 h to allow recovery before live imaging.

### Rap1a activity assay

A GST pull-down assay was performed to measure the Rap1 activity using GST-RalGDS-RBD that preferentially bound to Rap1-GTP. Briefly, the assay employs a GST fusion protein (GST-RalGDS-RBD) pre-coupled to glutathione-agarose beads, and then incubated with the sample of cell lysate for 1 h at 4 °C. Elute glutathione-agarose beads -bound GTPase with SDS buffer. The eluted sample can then be analyzed by western blot.

### RhoA activity assay

RhoA activity was measured using a RhoA Activation Assay kit (Cytoskeleton, Denver, CO) according to the manufacturer’s instructions. Briefly, a cell lysate was incubated at 4 °C for 1 h with a GST fusion protein containing the Rho-binding domain of rhotekin (GST-RBD) immobilized on glutathione-Sepharose beads. After washing, the bead-bound proteins were fractionated by SDS‒PAGE and immunoblotted with an anti-RhoA antibody. The total cell lysate was also blotted with the anti-RhoA antibody to permit assessment of the fractional ratio of RhoA binding protein.

### Gene expression analysis by RT-qPCR.

Freshly isolated tissue or cultured cells were lysed in TriZol reagent (Invitrogen, CA, USA) and cDNA was synthesized using the HiScript® II Q Select RT SuperMix for qPCR kit (Vazyme Biotech Co., Ltd, Nanjing, China). Quantitative RT–qPCR was performed in duplicate using FastStart Universal SYBR Green Master Mix (Roche Applied Science, Mannheim, Germany) using the StepOne real-time PCR System (Life Technologies Corp. Waltham, MA, USA). Expression levels are given relative to GAPDH. Sequences of primers are provided in Additional file 1: Table S1.

### Total, nuclear and cytoplasmic protein extraction

Total protein from cells or tissue was extracted according to the protein extraction kit (Vazyme, Nanjing, China). Nuclear and cytoplasmic proteins were extracted according to the Nuclear and Cytoplasmic Protein Extraction Kit (Sangon Biotech, Shanghai, China). The protein concentration was determined using a BCA protein assay kit (Va6zyme, Nanjing, China). β-actin and PARP were used to assess the purity of the cytoplasmic fraction and the nuclear fraction respectively.

### co-IP and western blot analysis.

Cells were washed once with ice-cold PBS and lysed in buffer containing Phos-stop phosphatase inhibitors and complete proteases inhibitors (Beyotime Biotechnology). The lysates were centrifuged at 15,000 rpm for 20 min at 4 °C, and the supernatant was used for immunoprecipitation. Wash Protein A + G magnetic beads with TBS buffer, and then incubated with anti-Rap1a and normal IgG at room temperature for 1 h. Wash beads complex with TBS buffer for three times. The cell lysates were incubated with the mixture overnight at 4 °C with rotation, whereafter beads were washed three times with lysis buffer and protein recovered by boiling samples for 10 min in sample buffer with 5% 2- mercaptoethanol. Proteins were eluted and subjected to SDS-PAGE. The protein was separated by SDS–PAGE (5%-12%), transferred onto 0.45 μm PVDF membranes (Solarbio, Beijing, China) and blocked in 10% nonfat milk in TBST for 2 h at room temperature. The blots were successively incubated with primary antibodies overnight at 4 °C. Following three washes with TBST, the membranes were then incubated with secondary antibodies at RT for 2 h. After three washes, the membranes were subjected to chemiluminescence using Clarity Western ECL Substrate (Affinity, Changzhou, China). Anti-β-actin was used as control. Protein expression was detected using an enhanced chemiluminescence detection system (ImageQuant LAS 4000 mini, USA). Antibody information is provided in Additional file 1: Table S2.

### Histological and immunohistochemical analysis

The uterus was fixed in 4% paraformaldehyde (PFA) solution and the tissue was embedded in paraffin. After fixation, tissue was washed 3 times with PBS and processed for the staining. Five micrometer mouse uterine sections were stained with haematoxylin and eosin (H&E) and stage of injury/repair graded by two masked independent observers using a previously published scoring system [54, 55]. Tissue sections were deparaffinized using xylene and rehydrated in graded ethanol. Sections were treated in 1 mM EDTA, pH 8.0 by boiling at 125 °C for 30 s and 90 °C for 10 s inside a cocker within a microwave for antigen retrieval. All sections were incubated with endogenous peroxidase with 1% H_2_O_2_ for 10 min and protein blocking reagents for 5 min each. The sections were then incubated with primary antibody and second antibody diluted in TBST diluent for 1 h at room temperature (RT). Afterwards, the sections were washed with TBS and then counterstained with hematoxylin. Images were obtained using an Imager Nikon Eclipse C1, a Nikon DS-U3 (Carl Zeiss) or an optical microscope (Olympus, Japan).

### TUNEL assay

TdT-UTP nick-end labelling (TUNEL) assay was performed a TUNEL assay kit (Roche Diagnostics GmbH) according to the manufacturer's instructions as described previously[56].

### Immunofluorescence

Cells grown on glass coverslips (also named sections) were fixed with 4% PFA for 15 min at room temperature (RT), washed 3 times with PBS and then permeabilized with 0.05% Triton X-100 for 10 min at RT. Paraffin embedded slides were dewaxed and rehydrated. Tissue sections were permeabilized with PBS containing 0.2% Triton X-100 before blocked with 10% normal goat serum for 1 h at RT. After washed 3 times with PBS, the sections stained with primary antibody in humidified chambers at 4 °C overnight. Following three 10 min washes in PBST, the sections incubated with secondary antibodies for 2 h at RT in the dark, phalloidin, depending on the staining, for 4 h at RT. Following three 5 min washes with PBST, the sections were stained with DAPI. Images were obtained using an Imager Nikon Eclipse C1 (Japan), a Nikon DS-U3 (Carl Zeiss) or an optical microscope (Olympus, Japan). For quantification of YAP subcellular localizations and positive cells were counted in at least 5 fields of view using ImageJ software, and at least 2 samples per experimental group were analyzed.

### Statistics and reproducibility

The in vitro experiments were repeated at least three times unless stated otherwise. As indicated in the figure legends, all quantitative data are presented as the mean ± S.D. or mean ± S.E.M. of at least three biologically independent experiments or samples. Statistical analyses were performed using GraphPad Prism 8 and Excel. Statistical significance was tested using an unpaired Student’s t-test or one/two-way ANOVA with Sidak’s multiple-comparisons test. ANOVA was used to compare more than two groups. All data were considered statistically significant at *P < 0.05; **P < 0.01.

## Supplementary Information

**Additional file 1.** Supplement Figure S1–S6 and Supplement Table S1–S2.

## Data Availability

The main data supporting the results in this study are available within the paper and its Supplementary Information. All the other materials are available from the corresponding author upon reasonable request.
