# Supplementary material for: Extracellular matrix stiffness mediates uterine repair via the Rap1a/ARHGAP35/RhoA/F-actin/YAP axis
Source: Cell Commun Signal. 2023 Jan 23;21:22. doi: 10.1186/s12964-022-01018-8 (PMC9869517; doi:10.1186/s12964-022-01018-8)
Supplement: Supplementary file 2 — Additional file 1. Supplement Figure S1–S6 and Supplement Table S1–S2. [file 12964_2022_1018_MOESM2_ESM.docx]

**Extracellular matrix stiffness mediates uterine repair via the Rap1a/ARHGAP35/RhoA/F-actin/YAP axis**

Tao Zhang*^#1,2^, Ruiting Hu^#1^, Yan Wang^1^, Shuai Guo^2^, Zhimin Wu^2^, Junfeng Liu^2,3^, Chunyang Han^1^, Changwei Qiu^2^, Ganzhen Deng^2^

^1^ College of Animal Science and Technology, Anhui Agricultural University, Hefei 230031, People’s Republic of China.

^2^ Department of Clinical Veterinary Medicine, College of Veterinary Medicine, Huazhong Agricultural University, Wuhan 430070, People’s Republic of China.

^3^ College of Animal Science and Technology, Tarim University, Alar, Xinjiang, 843300, People's Republic of China.

*** Correspondence**

Tao Zhang, College of Animal Science and Technology, Anhui Agricultural University, Hefei 230031, People’s Republic of China.

**E-mail:** [**taozhang@ahau.edu.cn**](mailto:taozhang@ahau.edu.cn)

Ganzhen Deng**,** Department of Clinical Veterinary Medicine, College of Veterinary Medicine, Huazhong Agricultural University, Wuhan, 430070, People's Republic of China.

E-mail: dgz@mail.hzau.edu.cn.

^#^ Contributed equally.

**Contents**

Supplement Figure S1-S6

Supplement Table S1- S2

**Supplement Figure**

**Figure S1**


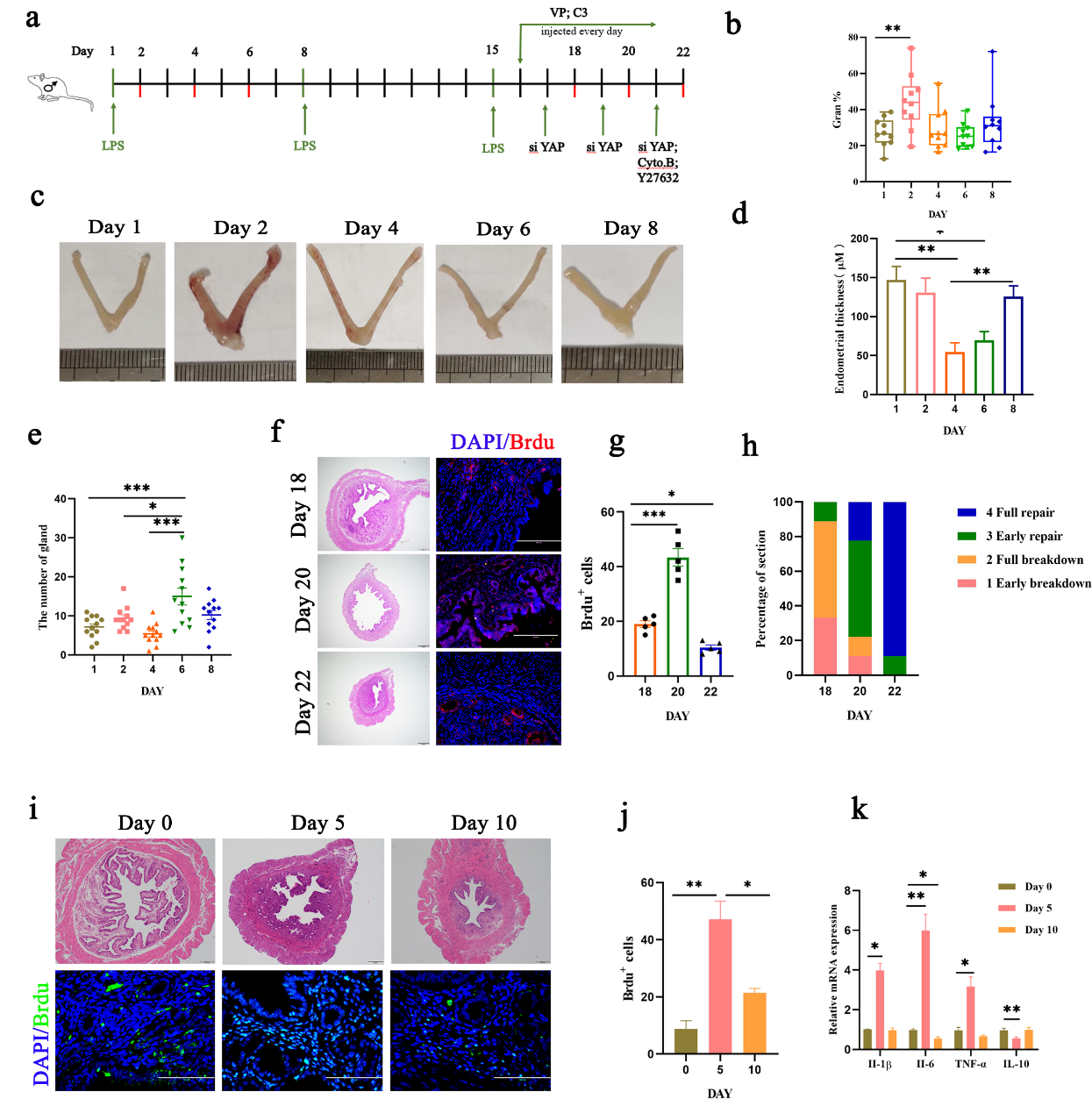


**Fig. S1 Endometrial injury and recovery in inflammatory-infected and postpartum mice. a.** Schematic diagram of the establishment of a mouse model of uterine injury and the timing of drug/agent administration. Inflammatory damage to mouse uterus tissues and blood were collected at 1, 2, 4, 6, and 8 d after injection of LPS. Postpartum injury to mouse uterus tissues and blood were collected at 0, 5, and 10 d. **b.** Percentage of neutrophils was analyzed by blood routine examination. n = 10. **c.** Representative whole uterus images. **d, e.** The number of endometrial glands and the thickness of the endometrium in different periods after injury. **f, g.** The uterine tissue collected for mice with 7 days after the last injection of three repetitive LPS injuries at 1-week intervals. Representative HE images and immunofluorescence images for Brdu of mouse uterine sections (**f**, n =2). Scale bar: 400 μm (left), 200 μm (right). Positive cells were enumerated in each uterine section (**g,** n = 5). **h.** Percentage of sections encompassing each of morphological stages at Day 18, Day 20 or Day 22 (**h**, n=6). **i-k.** Uterine tissues were collected on day 0, day 5, and day 10 of postpartum mice. Representative HE images and immunofluorescence images for Brdu of mice uterine sections (**i**, n =2). Scale bar: 400 μm (up), 100 μm (down). Positive cells were enumerated in each uterine section (**j,** n = 5). mRNA levels of IL-6, IL-1β and TNF-α in the endometrium were measured by RT-qPCR (**k**, n = 3). The data are presented as mean ± s.e.m. The experiments were repeated n times with duplicate biological replicates. *P < 0.05, **P < 0.01; ***P < 0.001.


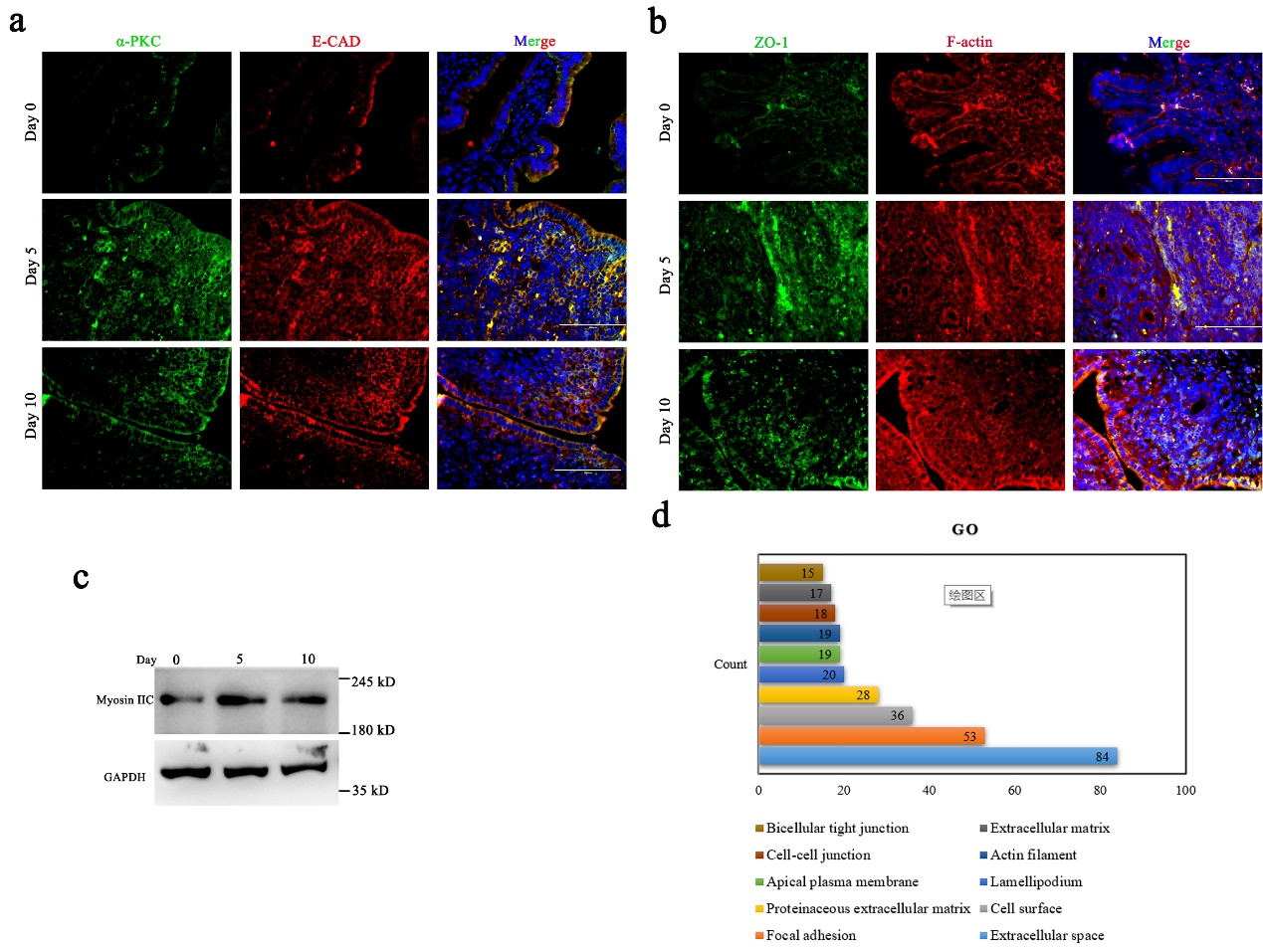


**Fig. S2 Changes in biomechanical cues in the postpartum mouse endometrium. a, b.** Immunofluorescence images of α-PKC and E-cad (**a**), and F-actin labeled by phalloidin and ZO-1(**b**) in the mouse uterine sections. Scale bar: 100 μm. n = 2. **c.** Lysates of uterine tissue obtained at different repair phases were analyzed for the presence of the indicated proteins. n = 3. **d.** Identification of pregnancy bovine endometrium DEGs from GEO dataset GSE40312. P< 0.01, |log FC| > 2. The Top 10 GO (biological process) term analyses of differentially expressed genes of the day 0 VS 15 after postpartum.


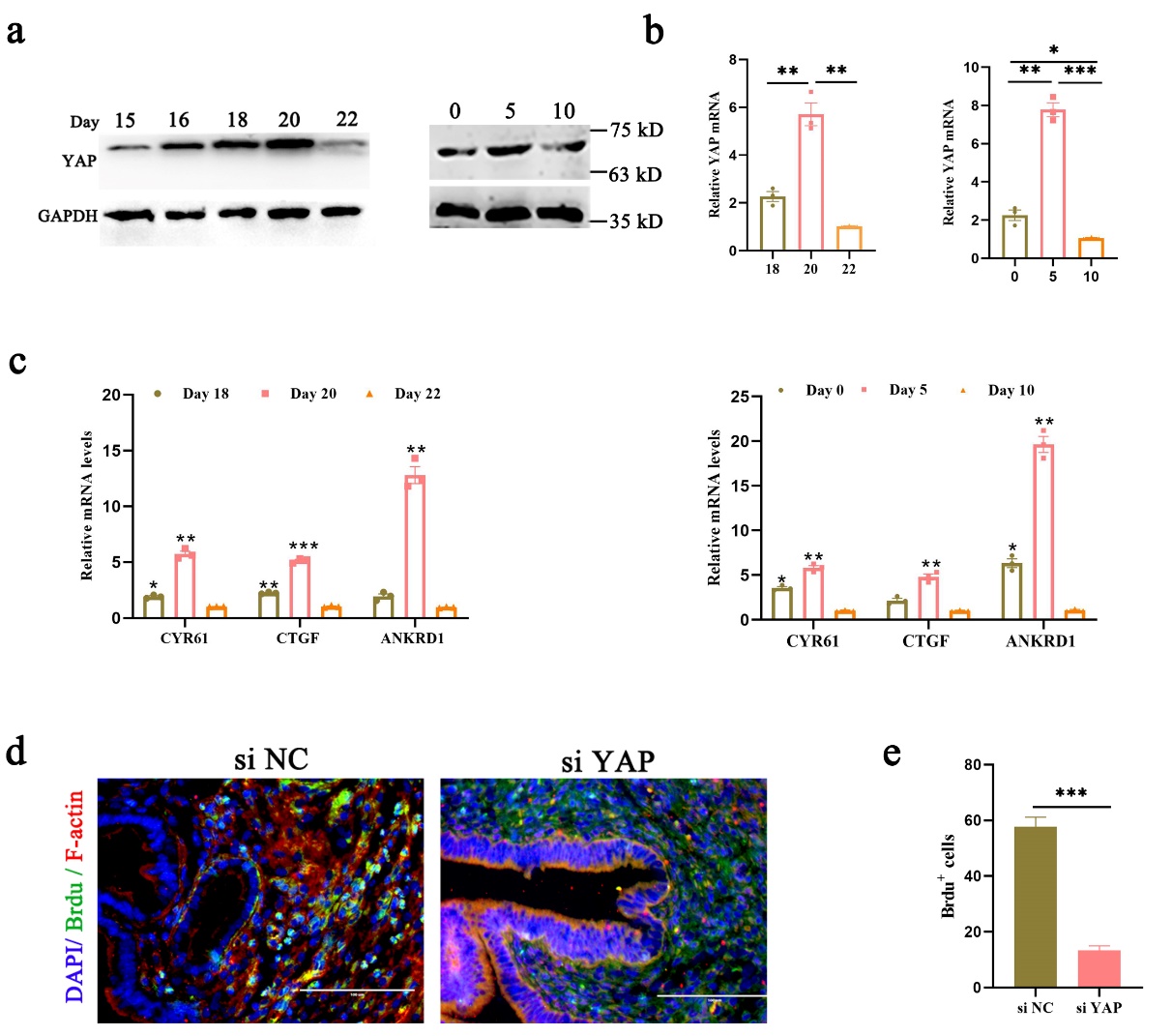


**Fig. S3 YAP is involved in uterine repair after multiple forms of injury. a.** Lysates of uterine tissue obtained at different repair phases were analyzed for the presence of the indicated proteins. n = 3. **b, c.** RT‒qPCR analysis of YAP (**b**), CYR61, CTGF and ANKRD1 (**c**) mRNA levels in uterine tissue for different repair phases. n=3. **d, e.** Immunofluorescence images of BrdU and F-actin (d) and quantification of BrdU+ positive cells, n = 2. Scale bars, 100 μm. The data are presented as mean ± s.e.m. The experiments were repeated n times with duplicate biological replicates. *P < 0.05, **P < 0.01; ***P < 0.001.


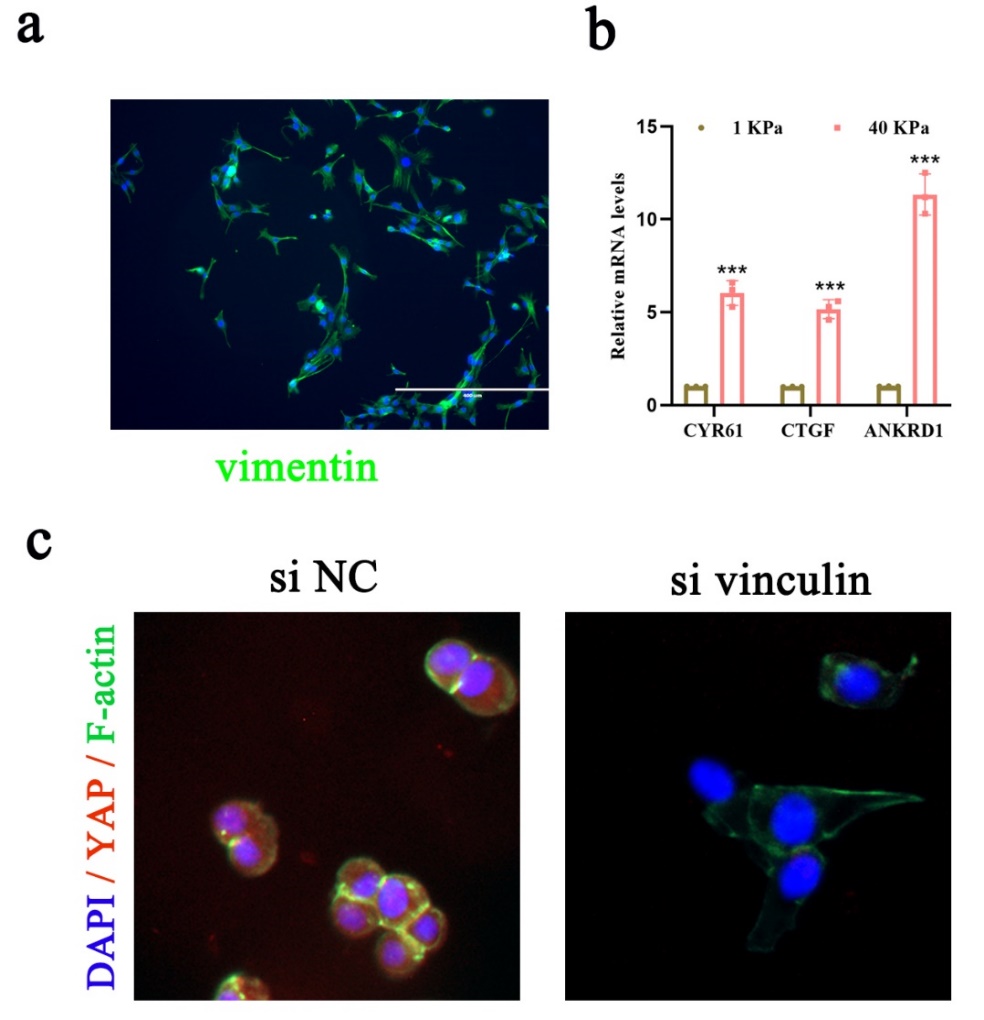


**Fig. S4 ECM stiffness regulates the activity of YAP in dependent focal adhesion in ESCs. a.** Immunofluorescence images of vimentin. Scale bars: 200 μm. n =2. **b.** RT‐qPCR analysis of CYR61, CTGF and ANKRD1 mRNA levels in ESCs. n = 3. **c.** Representative immunofluorescence of YAP in ESCs transfected with si vinculin at 40 kPa. Scale bars: 10 μm. n = 2. The data are presented as mean ± s.e.m. The experiments were repeated n times with duplicate biological replicates. *P < 0.05, **P < 0.01; ***P < 0.001.


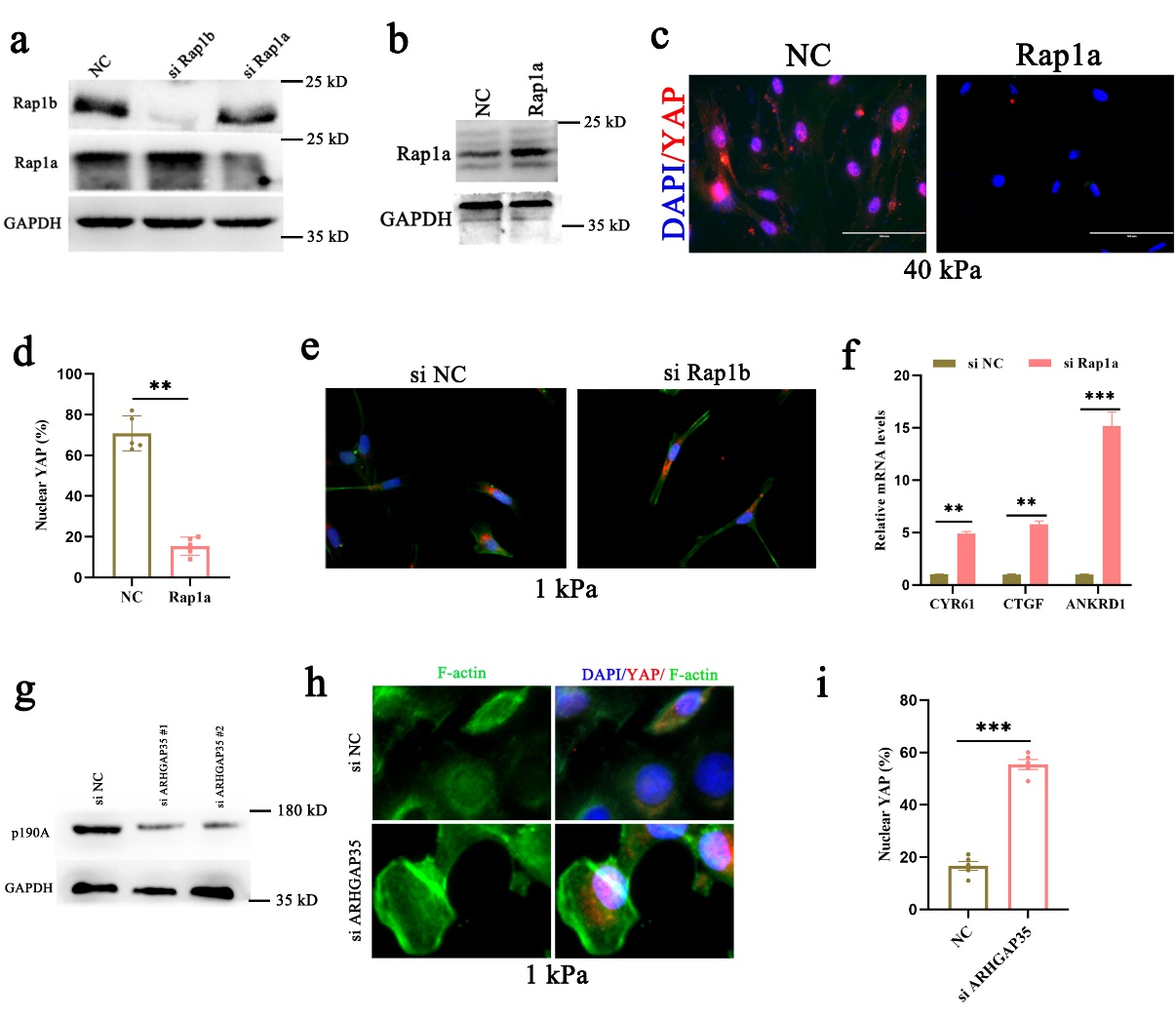


**Fig. S5 ECM stiffness induces YAP activation via the Rap1a/ARHGAP35 pathway. a, b.** Lysates of ESCs treated with si Rap1a, Rap1b or pcDNA3.1+Rap1a were analyzed for the presence of the indicated proteins. n =3. **c, d.** Confocal immunofluorescence images of YAP (**c**, n = 2) in ESCs transfected with pcDNA3.1+Rap1a or NC planted on 40 kPa hydrogel, and quantification of YAP+ positive cells was performed (**d**, n =5). Scale bars: 20 μm. **e.** Confocal immunofluorescence images of YAP in ESCs transfected with si Rap1b or NC planted on 1 kPa hydrogel. n = 2. Scale bars:10 μm. **f.** RT‒qPCR analysis of CYR61, CTGF and ANKRD1 mRNA levels in ESCs treatment with si Rap1a. n = 3. **g.** Lysates of ESCs treated with si ARHGAP35 #1/#2 were analyzed for the presence of the indicated proteins. n =3. **h, i.** Confocal immunofluorescence images (**h**, n = 2) and quantification of the nuclear and cytoplasmic subcellular localization (**i**, n = 5) of YAP in ESCs transfected with siARHGAP 35. Scale bar: 10 μm. The data are presented as mean ± s.e.m. The experiments were repeated n times with duplicate biological replicates. *P < 0.05, **P < 0.01; ***P < 0.001.


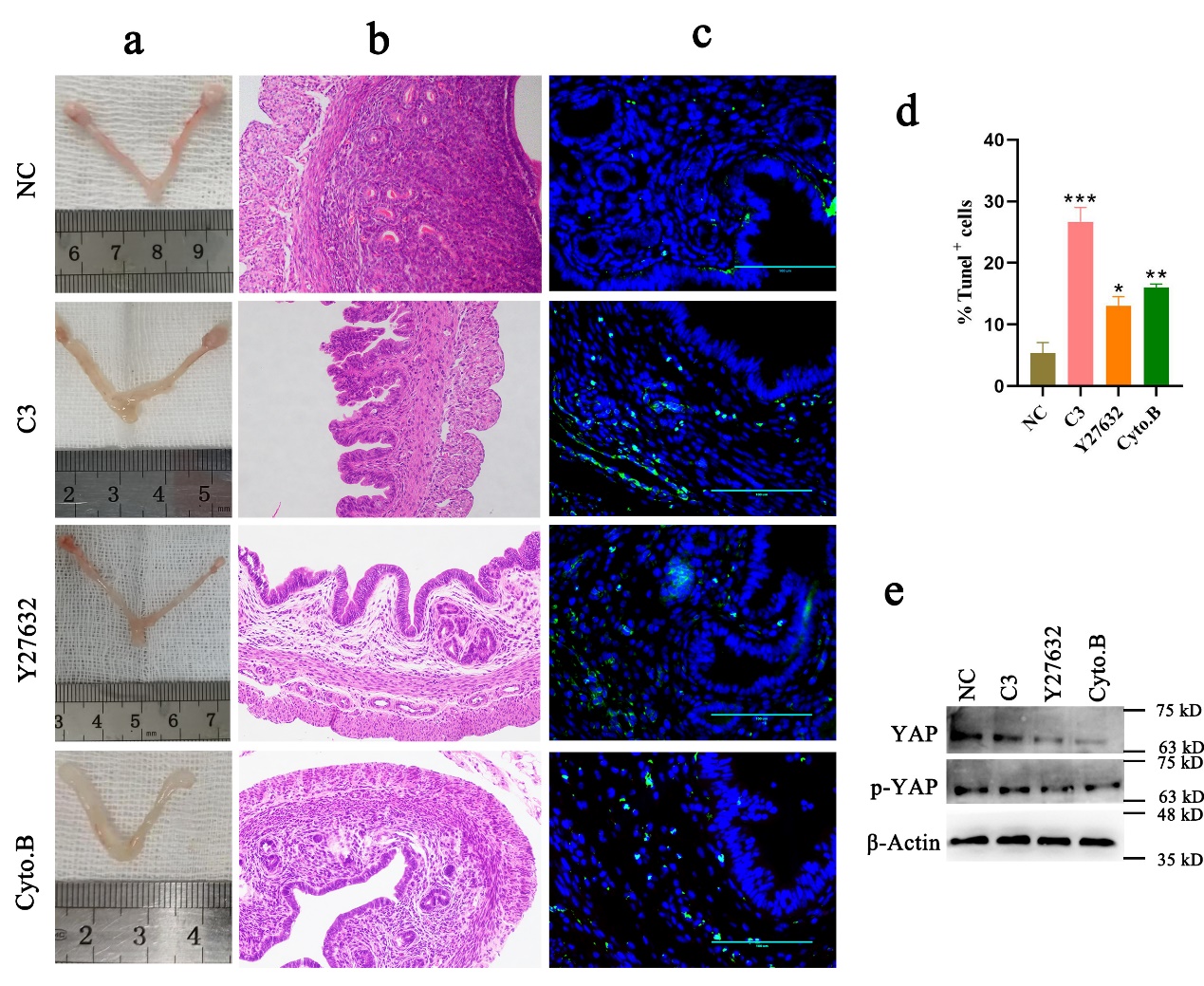


**Fig. S6 Blocking mechanotransduction prolongs the time required to repair the endometrium.** Uterine tissues were collected in inflammatory infected treated with C3, Y27632 and Cyto.B at day 20. **a, b.** Whole uterus images and uterine sections stained with H&E. Scale bars, 200 μm. **c, d.** Apoptosis in uterine sections was examined by TUNEL staining. n =2. Scale bar: 100 μm. **e.** Lysates of uterine tissue were analyzed for the presence of the indicated proteins. n =3. The data are presented as mean ± s.e.m. The experiments were repeated n times with duplicate biological replicates. *P < 0.05, **P < 0.01; ***P < 0.001.

| **Supplementary Table 1 oligonucleotide primers used for qPCR.** | | |
| --- | --- | --- |
| **Gene** | **Forward** | **Reverse** |
| YAP | GCGGTTGAAACAACAGGAATTA | TGAGACATCCCAGGAGAAGA |
| IL-6 | TTCCATCCAGTTGCCTTCTTG | CATTTCCACGATTTCCCAGAGA |
| IL-1β | GCAGCAGCACATCAACAAGA | GTTCATCTCGGAGCCTGTAGT |
| IL-10 | CAGGGATCTTAGCTAACGGAAA | GCTCAGTGAATAAATAGAATGGGAAC |
| TNF-α | ACTGGCAGAAGAGGCACTC | GGCTACAGGCTTGTCACTC |
| CYR61 | AGCCTCGCATCCTATACAACC | TTCTTTCACAAGGCGGCACTC |
| CTGF | CCAATGACAACGCCTCCTG | TGGTGCAGCCAGAAAGCTC |
| ANKRD1 | GTGTAGCACCAGATCCATCG | CGGTGAGACTGAACCGCTAT |
| GAPDH | CCATGTTCGTCATGGGTGTG | CAGGGGTGCTAAGCAGTTGG |

| **Supplementary Table 2. Information on antibodies used in the study** | | | | |
| --- | --- | --- | --- | --- |
| **Antibodies** | **Product code** | **Dilution** | **Species** | **Manufacturer** |
| GAPDH | #5174 | 1:1000 (WB) | Rabbit | Cell Signaling Technology |
| Anti-rabbit IgG | #5127 | 1:2000 (WB) | mouse | Cell Signaling Technology |
| Anti-mouse IgG | #7056 | 1:2000 (WB) | Goat | Cell Signaling Technology |
| YAP | #14074 | 1:1000 (WB), 1:250 (IF) | Rabbit | Cell Signaling Technology |
| YAP | ab81183 | 1:1000 (WB), 1:100 (IF) | Rabbit | abcam |
| Ki67 | ab15580 | 1:100 (IF) | Rabbit | abcam |
| p190A | ab247123 | 1:100(IF) | Rabbit | abcam |
| p190A | ab85950 | 1:1000(WB) | Rabbit | abcam |
| Lamin B1 | ab133741 | 1:5000(WB) | Rabbit | abcam |
| Myosin IIC | DF4196 | 1:500 (WB) | mouse | Affinity |
| Rap1a | A0975 | 1:1000 (WB) | Rabbit | Abclonal |
| Rap1b | A12925 | 1:1000 (WB) | Rabbit | Abclonal |
| Vinculin | A14193 | 1:1000 (WB), 1:50 (IF) | Rabbit | Abclonal |
| Caspase3 | A2156 | 1:100(IF) | Rabbit | Abclonal |
| CD45 | A2115 | 1:100(IHC) | Rabbit | Abclonal |
| CD11b | A1581 | 1:100(IHC) | Rabbit | Abclonal |
| Brdu | A1482 | 1:200(IF) | mouse | Abclonal |
| α-PKC | sc-8393 | 1:50(IF) | mouse | Santa Cruz Biotechnology |
| ZO-1 | sc-33725 | 1:100(IF) | mouse | Santa Cruz Biotechnology |
| E-cadherin | sc-8426 | 1:1000 (WB), 1:200 (IF) | mouse | Santa Cruz Biotechnology |
| YAP | sc-376830 | 1:1000 (WB), 1:50 (IF) | mouse | Santa Cruz Biotechnology |
| LATS1 | sc-398560 | 1:500 (WB) | mouse | Santa Cruz Biotechnology |
| Vimentin | GB12192 | 1:250 (IF) | mouse | Servicebio |
| Cy3 anti-rabbit IgG | GB21303 | 1:100 (IF) | Goat | Servicebio |
| Cy3 anti-mouse IgG | GB21301 | 1:100 (IF) | Goat | Servicebio |
| FITC anti-mouse IgG | GB22301 | 1:50 (IF) | Goat | Servicebio |
| FITC anti-rabbit IgG | GB22303 | 1:50 (IF) | Goat | Servicebio |
| FITC Phalloidin | G1028 | 1:200 (IF) |  | Servicebio |
| TRITC Phalloidin | G1041 | 1:200 (IF) |  | Servicebio |
